# Comprehensive synthesis and characterization of a novel Fe–porphyrin complex: crystal structure, spectroscopic investigations, Hirshfeld surface analysis, and computational modeling (DFT, QTAIM-NCI/ELF)

**DOI:** 10.3389/fchem.2025.1666671

**Published:** 2025-09-15

**Authors:** Mondher Dhifet, Lelfia Guelmami, Khadija Zaki, Imen Zghab, Abdelouahid Sbai, Bouzid Gassoumi

**Affiliations:** 1 Laboratory of Physico-Chemistry of Materials (LR01ES19), Faculty of Sciences of Monastir, avenue of Environment, Monastir, Tunisia; 2 Faculty of Sciences of Gafsa, University of Gafsa, Gafsa, Tunisia; 3 Laboratory of Functional Physiology and Valorization of Bio-Resources (UR17ES27) at the Higher Institute of Biotechnology of Beja (ISBB), University of Jendouba, Jendouba, Tunisia; 4 National Institute of Technology and Sciences of Kef, University of Jendouba, Jendouba, Tunisia; 5 Laboratory of Molecular Chemistry and Natural Substances, Faculty of Sciences, University of Moulay Ismail, Meknes, Morocco; 6 Department of Physical Sciences, Chemistry Division, College of Science, Jazan University, Jazan, Saudi Arabia; 7 Laboratory of Advanced Materials and Interfaces (LIMA), University of Monastir, Faculty of Sciences of Monastir, Avenue of Environment, Monastir, Tunisia

**Keywords:** iron(II)–porphyrin, X-ray diffraction, Fourier-transform infrared, UV–visible, density functional theory, electron localization function–localized orbital locator

## Abstract

In this work, we prepared the novel (η^1^-hydrogencarbonato) iron (II) picket-fence porphyrin with the formula [K (2,2,2-crypt)][Fe^II^(TpivPP) (η^1^-HCO_3_)] (I) (where TpivPP is the (α,α,α,α-tetrakis (*o*-pivalamidophenyl) (porphinato) anion and (2,2,2-crypt) is cryptand-2,2,2). Complex I was characterized by UV–VIS and IR spectroscopy and single-crystal X-ray diffraction(XRD). These techniques show that the HCO_3_^−^ axial ligand is coordinated to the Fe^2+^ metal ion in a monodentate mode. Complex I crystallizes in the *P2*_*1*_ */n* space group with one ion complex [Fe^II^(TpivPP) (HCO_3_)]^-^ and one counterion [K (2,2,2-crypt)]^+^. The average equatorial iron–pyrrole nitrogen [Fe–N_p_ = 2.079 (3) Å] bond length and the distance between the iron atom and the 24-atom core of the porphyrin ring [Fe–P_C_ = 0.466 (1) Å] are comparable to those of other five-coordinate, high-spin (S = 2) iron (II) porphyrinates. This is probably due to the electronic repulsion between the d_x_^2^_-y_^2^ and d_xy_ orbitals and the negative charge of the pyrrole nitrogen. To complement these structural insights, density functional theory (DFT) calculations were performed on the individual ionic components to elucidate their intrinsic electronic properties and reactivity. The molecular electrostatic potential (ESP) maps clearly demonstrated the expected charge complementarity, with a globally positive surface for the [K (2,2,2-crypt)]^+^ counterion and a predominantly negative potential—localized notably on the oxygen atoms of the η^1^-hydrogencarbonato ligand—for the [Fe^II^(TpivPP) (HCO_3_)]^−^ ion complex. Analysis of the frontier molecular orbitals further revealed the electron-donating propensity of the porphyrin π-system and the axial ligand in the anion, juxtaposed with the electron-accepting capability of the cation. Finally, Hirshfeld surface analysis provided detailed insights into the intermolecular interactions within the crystal lattice, highlighting significant non-covalent contacts, including various hydrogen bonds, which govern molecular packing and contribute to the overall crystal stability. This combined experimental and computational approach offers a comprehensive understanding of the structural, electronic, and intermolecular features of this novel iron (II)–porphyrin complex.

## 1 Introduction

The iron metalloporphyrins are used as models for certain hemoproteins. The active sites of hemoglobin and myoglobin are of considerable biological significance (Dhifet et al., 2009a; Dhifet et al., 2024; Huang and Groves, 2017; Moreira et al., 2015). In cases where the iron coordination number is six, the high-spin state has led many authors to conclude that this spin state is not always possible.

Many X-ray crystal structures of bidentate axial ligand (*η* ^2^-hydrogencarbonato) porphyrin complexes have been reported since 1970, with TPP (tetraphenylporphyrin) (Tutass et al., 2002) and one with STTP (5, 10, 15, 20-tetratolyl-21-thiaporphyrinato) (Szyszko and Latos‐Grażyński, 2020). The study of these species was specifically aimed at understanding the oxo-transfer reactions involved in many catalytic organic reactions (Ali et al., 2011). It has been shown that the high spin state (S = 2) can fall into two different electronic configurations: (i) the first type is the (d_xy_)^2^ (d_xz_)^1^ (d_yz_)^1^ (d_z_^2^)^1^ (d_x_^2^_-y_^2^)^1^ derivatives of type [Fe^II^(Porph) (X)]^-^ (Porph = porphyrin, and X is a monodentate anionic weak field ligand), and (ii) the second type is (d_xz_)^2^ (d_yz_)^1^ (d_xy_)^1^ (d_z_^2^)^1^ (d_x_^2^_-y_^2^)^1^ with the formula [Fe^II^(Porph) (X)_2_] (where X is a monodentate neutral σ-donor ligand) (Hu et al., 2010).

The crystal structure of (*η* ^1^-hydrogencarbonato) iron (II) has not been reported in the literature.

In the Cambridge Structural Database updated to November 2023 (CSD 2023 2.0, version 5.45) (Groom et al., 2016), only two metalloporphyrins with a hydrogencarbonato axial ligand (HCO_3_^−^) with the formulas [Mg^II^(TPP) (η^1^-HCO_3_)]^-^ (Bhuyan et al., 2011) and [Zr^IV^(TPP) (η^1^-HCO_3_) (OPh)] (Dhifet et al., 2025) are reported.

To better understand the molecular structure and electronic, magnetic, and spectroscopic properties of complex I with the monodentate axial ligand (η^1^-hydrogencarbonato), it is necessary to use the picket-fence porphyrin (H_2_TpivPP). The use of H_2_TpivPP is associated with the high stability of complexes containing iron (II)-bound anionic ligands on the protected side of the porphyrin and helps prevent the formation of *μ*-oxo complexes.

To gain further insights into the hydrogencarbonate porphyrin complexes, this work reports the synthesis, UV–VIS and IR spectroscopic properties, and the single-crystal X-ray molecular structure of a new five-coordinate, high-spin (S = 2) iron (II) hydrogencarbonato porphyrinic coordination compound. Our compound is similar to [K (2,2,2-crypt)][Fe^II^(TpivPP) (OAc)] (Salhi et al., 2025). The intermolecular interactions in the crystal lattice of complex I were studied by Hirshfeld surface analysis, performed using the PLATON program.

Furthermore, to gain a deeper understanding of the electronic structure, charge distribution, and intrinsic reactivity of the individual ionic components, we performed density functional theory (DFT) calculations on both the cationic and the anionic fragments. These computational studies, including analyses of the molecular electrostatic potential (MEP), frontier molecular orbital (FMO), electron localization function (ELF), and localized orbital locator (LOL) maps analyses, provide essential insights into the respective electron-donating and -accepting capabilities of the components and reveal the complementarity of their electronic landscapes, which governs their interactions and contributes to the stability of the overall complex.

## 2 Experimental section

### 2.1 General procedures

All the experiments were conducted under an inert atmosphere using the Schlenk technique. Chlorobenzene was purified by washing with sulfuric acid and then distilled over P_2_O_5_, and *n*-hexane was distilled over CaH_2_. KHCO_3_ was recrystallized twice from distilled water, dried overnight at approximately 70 °C, and stored under argon. The cryptand-2,2,2 was recrystallized from toluene (dried by distillation over sodium/benzophenone) and stored under argon in the dark. All other chemicals were commercially available and used as received without further purification.

Electronic spectra were recorded on a Shimadzu UV-2401 Spectrometer - Mundelein, IL, United States (World News of Natural Sciences 31 (2020) 155-174). and Shimadzu FT-IR-8400 Spectrometer - The establishment of EVISA is funded by the EU through the Fifth Framework Programme (G7RT- CT- 2002- 05112)..

Hirshfeld surface (HS)/2D fingerprint plot analyses were performed using Crystal Explorer 3.15 (Espíndola and Gázquez, 2023; Wolff et al., 2013). These tools provide insights into the inter- and intra-atomic interactions between the groups of complex I. The “*d*_*norm*_” Hirshfeld surface is a normalized distance generated as “*d*_*e*_” in the function “*d*_*i*_,” which visualizes all the intermolecular contacts of the crystal structure (Spackman and Jayatilaka, 2009; Spackman and McKinnon, 2002).

### 2.2 Synthesis of [K (2,2,2-crypt)][Fe^II^(TpivPP) (η^1^-HCO_3_)]^•^C_6_H_5_Cl

The free-base picket-fence porphyrin (H_2_TpivPP) (Colman et al., 1975) and the corresponding iron (III) chloro and triflato (Gismelseed et al., 1990) derivatives were synthesized by literature methods. A sample of 100 mg (0.081 mmol) of [Fe^III^(TpivPP) (SO_3_CF_3_) (H_2_O)] was placed in a first Schlenk flask containing 10 mL of distilled chlorobenzene and 1 mL of zinc amalgam, and the mixture was stirred for 1 h at room temperature. The red solution of the obtained Fe(II) solution made by the [Fe^II^(TpivPP)] complex was then filtered into a second Schlenk flask containing 120 mg (0.319 mmol) of cryptand-2,2,2 and 190 mg (2.262 mmol) of KHCO_3_ in 10 mL of chlorobenzene. The mixture was stirred for 2 h and then filtered. The red solution was then filtered, and single crystals of the complex were prepared by slow diffusion of hexane through the chlorobenzene solution.

Elemental analysis was calculated for C_89_H_106_ClFeN_10_KO_13_ (molecular weight = 1654.25), yielding C, 64.61%; H, 6.47%; and N 8.46%; the found values were C, 64.65%; H, 6.61%; and N, 9.08%. The UV–VIS spectrum showed absorption maxima at (λ_max_) (nm, C_6_H_5_Cl, logε) 440 (5.86), 568 (4.61), 612 (4.51), and 632 (4.01). The IR spectrum exhibited bands at (cm^-1^) 3,403 (s), 2,960–2,871 (s), 1,680 (s), 1,641 (w), 1,356 (s), 1,102 (vs.), 987 (s), and 751(s).

### 2.3 X-ray structure determination

The single crystal of the iron (II) hydrogencarbonato porphyrin derivative (I) with dimensions of 0.31 × 0.22 × 0.18 mm^3^ was collected at room temperature on a Bruker-AXS APEX2 2014 with Mo Kα radiation (λ = 0.71073 Å). The structure was solved by the direct methods using the program SIR-2004-1.0 (Burla et al., 2005) in the space group *P2*_*1*_ */n*, with four formulas per unit cell. The structure refinement was conducted against *F* ^*2*^ data using the program SHELXL-97 (Sheldrick, 2008). The residual electron density arising from several disordered solvent molecules in the void space could not be satisfactorily modeled; therefore, these solvent molecules were removed using the SQUEEZE function of the PLATON program (Spek, 2015; Spek, 2020). A total of 96 electrons were accounted for by the SQUEEZE function and removed. This amounts to one chlorobenzene and four water molecules (confirmed by the CHN elemental analysis) by the asymmetric unit of the structure. During the refinement procedure of complex I, one tert-butyl of the pivalamide is disordered over two positions [C50-C51A-C52A-C53A and C50-C51B-CC52B-C53B] with occupancy percentages of 73.3% and 26.7%, respectively.

The anisotropic displacement ellipsoids (ADPs) of the one disordered tert-butyl of the pivalamide group and the atoms of the hydrogenocarbonato axial ligand are very elongated. Thus, for these atoms, the DELU, SIMU, and SADI constraint commands in SHELXL-97 software were used. The DFIX constraint command was also used to correct the geometry of the hydrogenocarbonato axial ligand (McArdle, 1995). Drawings were made using ORTEP-3 for Windows (Farrugia, 2012) and MERCURY (Macrae et al., 2008). The crystallographic data and structural refinement details of complex I are shown in Table 1, and selected bond lengths and angles for this new iron (II) metalloporphyrin are listed in Table 2.

**TABLE 1:** Crystal data and structural refinement for complex I *.

Empirical formula	C_83_H_101_FeKN_10_O_13_
Formula weight, M	1,541.68
T (K)	293 (2)
Wavelength (Å)	0.71073
Crystal system	Monoclinic
Space group	*P2*_*1*_ */n*
a (Å)	18.162 (3)
b (Å)	21.612 (3)
c (Å)	22.604 (6)
β (°)	100.34
Volume V (Å3)	8,728.4 (17)
Z	4
Dcal, (g/cm^3^)	1.173
Extinction coefficient	0.030 (5)
Absorption coefficient, μ (mm^-1^)	0.283
F (000)	3272
Crystal size (mm^3^)	0.31 × 0.22 × 0.18
θ Range for data collection	2.06–26.00
Limiting indices	−21 ≤ h ≤ 19, −24 ≤ k ≤ 26, and −18 ≤ l ≤ 27
Reflections collected/unique	15,411/11,115
R (int)	0.1107
Absorption correction	multi-scan
Maximum and minimum transmission	0.954 and 0.865
Refinement method	Full-matrix least-squares on F2
Data/restraints/parameters	11115/123/948
Final R indices [I > 2σ(I)]	R1^a^ = 0.1055 and wR2^b^ = 0.2830
R indices (all data)	R1 = 0.1303 and wR2 = 0.3075
Goodness-of-fit on Fo2	1.048
Highest peak and deepest hole (eÅ-3)	1.585 and −0.900
CCDC	2,414,836

^a^ *R*_1_ = Σ||*F*o| – |*F*c||/Σ|*F*o|.

^b^ *wR*_2_ = {Σ[*w* (|*F*o|2 – |*F*c|2)2]/Σ[*w* (|*F*o|2)2]}1/2.

*Chlorobenzene removed using the squeeze procedure was not taken into account.

**TABLE 2:** Distances (Å) and angles (°) of the iron coordination polyhedron and the cation complex.

Iron coordination polyhedron					
Fe–N1	2.070 (3)	N1–Fe–N2	88.15 (13)	N1–Fe–O5	97.48 (16)
Fe–N2	2.055 (3)	N1–Fe–N3	157.33 (14)	N2–Fe–O5	104.65 (16)
Fe–-N3	2.083 (3)	N1–Fe–N4	86.67 (13)	N3–Fe-O5	105.04 (16)
Fe–N4	2.098 (3)	N2–Fe–N4	157.90 (14)	N4–Fe–O5	97.32 (16)
Fe–O5	2.070 (4)	N2–Fe–N3	88.65 (12)	C65–Fe–O5	123.5 (4)
Hydrogencarbonato ligand					
O5–C65	1.219 (4)	O6–C65–O5	117.5 (6)		
O6–C65	1.242 (4)	O6–C65–O7	118.6 (5)		
O7–C65	1.386 (5)	O5–C65–O7	123.9 (5)		
O7–HO7	0.8200				
Potassium-cryptand-222					
K–N9	3.024 (4)	K–O11	2.825 (3)	N9–K–O9	120.00 (12)
K–N10	3.054 (4)	K–O12	2.846 (3)	N9–K–O10	120.20 (11)
K–O8	2.801 (4)	K–O13	2.786 (4)	N9–K–O11	60.54 (11)
K–O9	2.820 (3)	N9–K–N10	179.84 (14)	N9–K–O12	60.86 (12)
K–O10	2.854 (3)	N9–K–O8	59.72 (13)	N9–K–O13	120.39 (12)

## 3 Results and discussion

### 3.1 Synthesis of complex I

**SCHEME 1:**
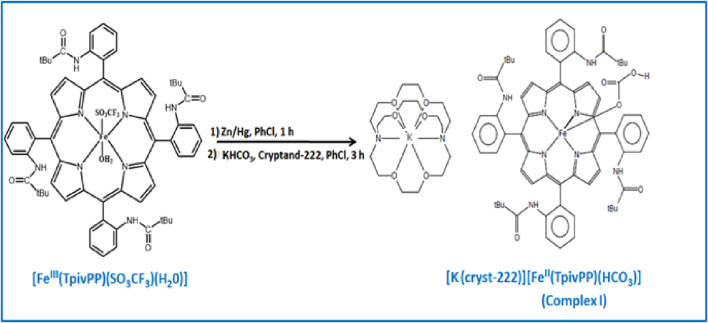
Synthetic procedure of complex I.

The free *meso-*porphyrin H_2_TpivPP and the corresponding iron (III) chloro and triflato (Colman et al., 1975; Gismelseed et al., 1990) were synthesized according to literature methods. The synthesis of the starting products (H_2_TpivPP, [Fe^III^(TpivPP)Cl], and [Fe^III^(TpivPP) (SO_3_CF_3_) (H_2_O)]) is illustrated in Supplementary Schemes S1–3. The different steps in the preparation of complex I are shown in Supplementary Figure S1 and the synthetic procedure is depicted in Scheme 1.

### 3.2 Spectroscopic investigations

**FIGURE 1:**
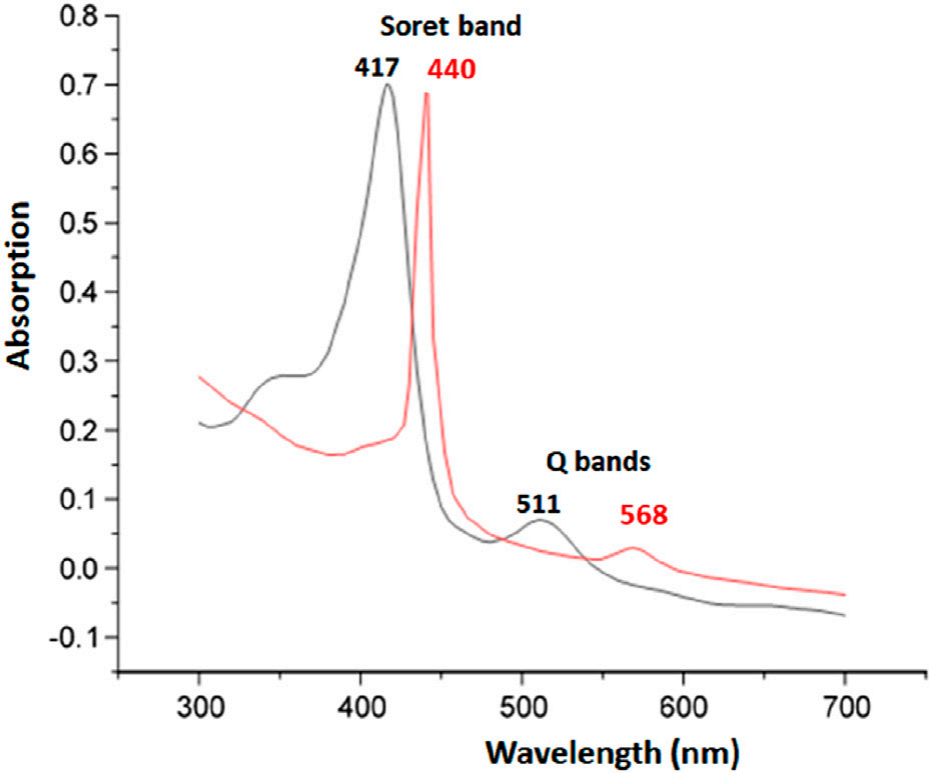
UV-visible absorption spectra of the [Fe^III^(TpivPP)(SO_3_CF_3_)(H_2_O)] (black) and the [K(2,2,2-crypt)][Fe^II^(TpivPP)(HCO_3_)] (red) recorded in C_6_H_5_Cl at concentrations.

Figure 1 depicts the spectra of complex I and the [Fe^III^(TpivPP) (SO_3_CF_3_) (H_2_O)] starting material.

The position of the Soret band at 440 nm in chlorobenzene clearly indicates a red shift. In such compounds, the strong red shift of the Soret band is attributed to charge effects arising from the negative charges of both the ligand and the complex ion.

The values of λ_Max_ corresponding to the maximum absorbance of the synthesized compound and other compounds are given in Table 3.

**TABLE 3:** Electronic spectra data^a^ for compound I and a selection of picket-fence Fe (II) five-coordinated ion complexes.

Complex	*λ*_max_ (nm)			Spin *S*	Reference
	Soret region	α and β regions			
[Fe^II^(TpivPP) (NO_2_)]^-^	444	567	608	0	Nasri et al. (1991a)
[Fe^II^(TpivPP) (CN)]^-^	455	565	601	0	Ben Abbes (2005)
[Fe^II^(TpivPP) (OAc)]^-^	448	572	611	2	Nasri et al. (1987)
[Fe^II^(TpivPP) (OMe)]^-^	456	580	622	2	Nasri et al. (1987)
[Fe^II^(TpivPP) (NO_3_)]^-^	438	564	604	2	Nasri et al. (2006)
[Fe^II^(TpivPP) (NCO)]^-^	437	568	610	2	Dhifet et al. (2010)
[Fe^II^(TpivPP) (N_3_)]^-^	443	572	612	2	Hachem et al. (2009)
[Fe^II^(TpivPP) (HCO_3_)]^-^	440	568	612	2	this work

^a^ Solvent: chlorobenzene.

As shown in Table 3, the Soret band of our compound at 440 nm is in the range [437 nm–455 nm], which characterizes iron (II) five-coordinate *meso*-arylporphyrin complexes with anionic axial ligands. It can be concluded that our synthesized complex I is very similar to the low-spin (S = 0) and high-spin (S = 2) penta-coordinated iron (II) porphyrin complexes. According to the UV–VIS results, compound I is an iron (II) five-coordinated porphyrin complex, but the spin state of the iron (II) is not confirmed; this can be confirmed by other characterization techniques. This UV–VIS investigation indicated that, in a solution, complex I is an iron (II) penta-coordinated picket-fence porphyrin complex.

The optical gap (E_g-opt_) values for complex I were calculated using the following formula and the tangent method (see Supplementary Figure S2):

$$E_{g-opt}=\frac{1240}{\lambda_{\mathrm{Lim}}}.$$

The experimental value is 2.016 eV, indicating the semiconductor character of our hydrogencarbonato iron (II) complex.

The IR spectra of the H_2_TpivPP free-base porphyrin and the [Fe^III^(TpivPP) (SO_3_CF_3_)] starting material are depicted in Supplementary Figure S3.

H_2_TpivPP exhibits a characteristic IR spectrum of a *meso*-arylporphyrin with ν(NH) and ν(CH) stretching frequencies at 3,430 cm^−1^ and in the range of 2,960 cm^−1^–2,869 cm^−1^, respectively.

The metalation of H_2_TpivPP with iron (II) chloride dihydrate and the addition of silver triflate in THF lead to the disappearance of the absorption band corresponding to the ν(NH) stretching and the shift toward the high fields of the absorption band attributed to δ(CCH) bending from 967 cm^−1^ to 987 cm^−1^.

**FIGURE 2:**
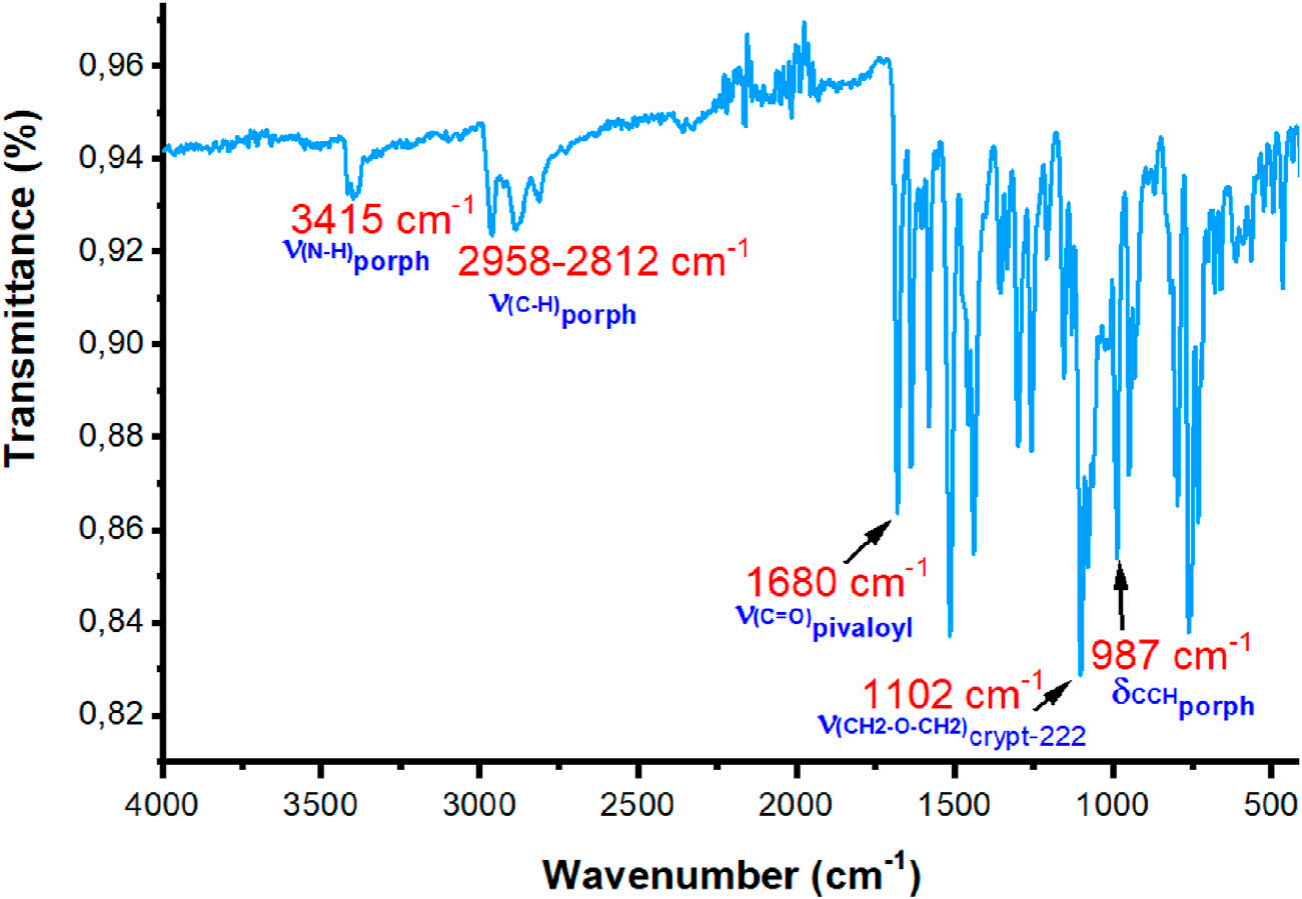
Experimental IR spectrum of complex I.

The experimental IR spectrum of complex I is shown in Figure 2.

The values of the sensitive bands of some porphyrin complexes of iron (II) with the porphyrin TpivPP are provided in Table 4.

**TABLE 4:** Values of the sensitive bands of some porphyrin complexes of iron (II) with the porphyrin TpivPP.

Complex	ν(NH)_porph_	ν(CO)_porph_	δ(CCH)_porph_	Reference
Iron (II) penta-coordinated				
[Fe^II^(TpivPP) (N_3_)]^-^	3408	1685	985	Hachem et al. (2009)
[Fe^II^(TpivPP) (CN)]^-^	3419	1683	988	Ben Abbes (2005)
[Fe^II^(TpivPP) (OMe)]^-^	3418	1685	985	Nasri et al. (1987)
[Fe^II^(TpivPP) (OAc)]^-^	3414	1682	989	Nasri et al. (1987)
[Fe^II^(TpivPP) (NO_3_)]^-^	3418	-	984	Nasri et al. (2006)
[Fe^II^(TpivPP) (HCO_3_)]^-^	3415	1680	987	This work
Iron (II) hexa-coordinated				
[Fe^II^(TpivPP) (NO_2_) (CO)]^-^	3421	-	996	Nasri et al. (2004)
[Fe^II^(TpivPP) (N_3_) (CO)]^-^	3405	-	992	Nasri et al. (1991b)

For our compound [K (2,2,2-crypt)][Fe^II^(TpivPP) (HCO_3_)], there is a strong band in the IR spectrum at 1,102 cm^-1^, which is attributed to the counterion (cryptand-2,2,2)potassium (+), and the δ(CCH) bending frequency value is 987 cm^−1^. The presence of a counterion and δ(CCH)_porph_ at 987 cm^-1^ confirmed the five-coordinate iron (II) porphyrin species with ionic axial ligands.

Notably, the low value of the δ(CCH) deformation frequency (988 cm^−1^) is characteristic of iron (II) penta-coordinated *meso*-arylporphyrins (887 < $\bar{\nu }$ < 990 cm^−1^) (Nasri, 1987). As shown in Table 4, this value is practically the same as that of the acetato iron (II)-related species [Fe^II^(TpivPP) (η^1^-OAc)]^-^ (Nasri et al., 1987). Regarding the HCO_3_^−^ axial ligand, seven out of eight frequencies were identified in the IR spectrum of the current compound.

The absorption bands assigned to the C–O of the hydrogencarbonate group are 1,641 cm^−1^ and 1,356 cm^−1^, respectively. The C–O theoretical bands appear at approximately 1,645 cm^−1^ and 1,354 cm^−1^ (see Supplementary Figure S4). The weak absorption band at 1,686 cm^−1^ is assigned to the C=O stretching frequency of the TpivPP porphyrin. The theoretical value of this band is approximately 1,684 cm^−1^. The DFT-D3 and experimental FT-IR results are consistent and exhibit a symmetrical and organized system. The UV–VIS and IR spectroscopies failed to provide a decisive answer or confirm the spin-state and ground-state electronic configuration of the central ion.

### 3.3 X-ray molecular structure of complex I

The different steps for preparing a crystal for X-ray diffraction (XRD) are shown in Supplementary Figure S5.

**FIGURE 3:**
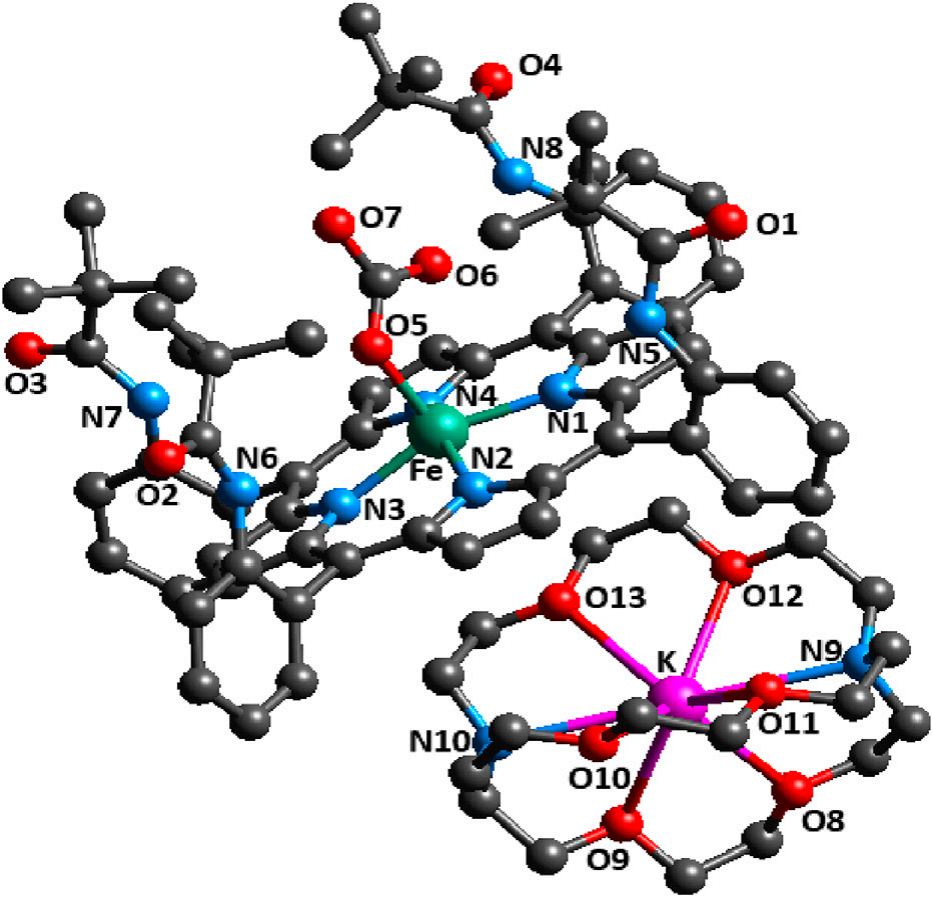
Molecular structure of the [K (2,2,2-crypt)][Fe^II^(TpivPP) (HCO3)]-ion complex.

The molecular structure of the [K (2,2,2-crypt)][Fe^II^(TpivPP) (HCO_3_)] ion complex is shown in Figure 3.

The iron atom is five-coordinated, with the one oxygen atom of the axial ligand HCO_3_^−^ and the four nitrogen atoms in the pocket porphyrin of H_2_TpivPP.

**FIGURE 4:**
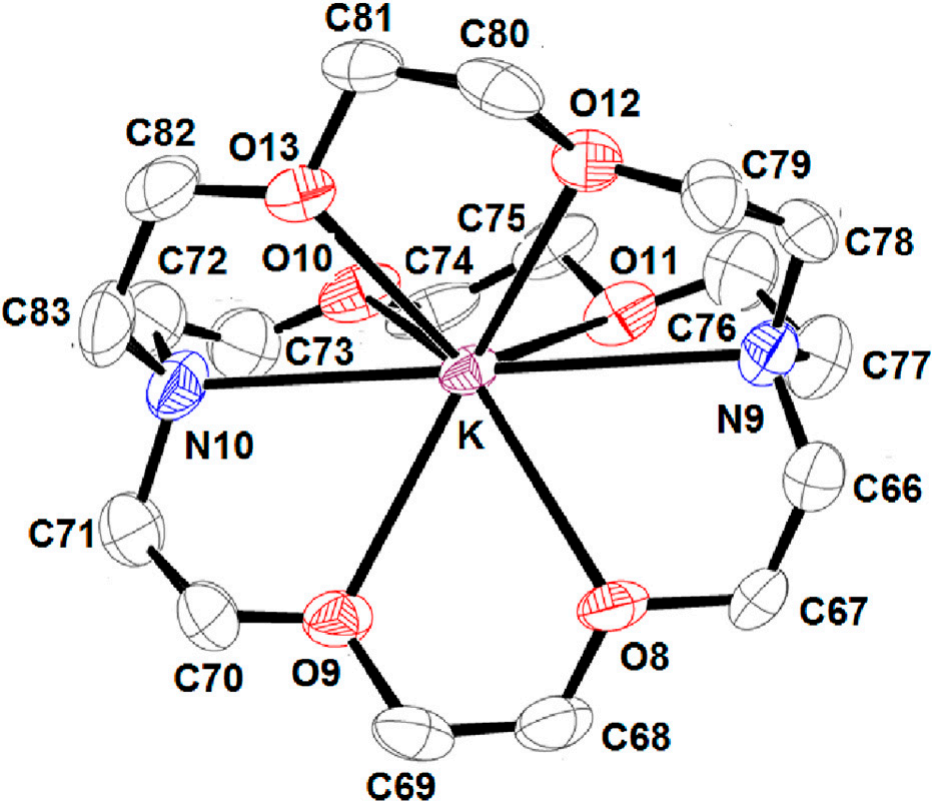
ORTEP view of the [K (2,2,2-crypt)]^+^ counterion, where the thermal ellipsoids are drawn at the 30% probability level. Hydrogen atoms have been omitted for clarity.

Figure 4 depicts an ORTEP diagram (Farrugia, 2012) of the [K (2,2,2-crypt)]^+^ ion complex.

**FIGURE 5:**
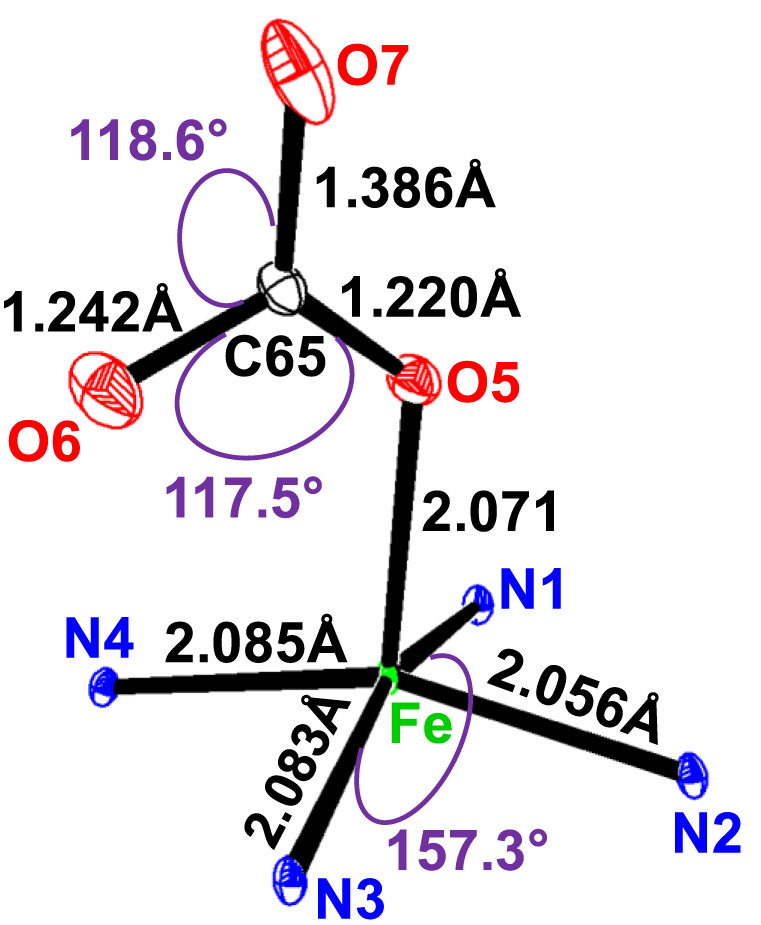
Selected distances (Å)/angles (°) of the iron coordination polyhedron.

The potassium cation is bonded to two nitrogen atoms and six oxygen atoms of the cryptand-2,2,2. The K–O (crypt-2,2,2) and K–N (crypt-2,2,2) distances [3.039 (4) Å and 2.822 (3) Å, respectively] are consistent with the values reported in the literature (Allen and Bruno, 2010). Figure 5 describes the coordination polyhedron of the central ion.

The geometric parameters of the Fe–Np, Fe–X_L_, and Fe–P_C_ distances of compound I and the other five-coordinate iron (II) are reported in Table 5.

**TABLE 5:** Stereochemical parameters (Å) for low-spin (S = 0) and high-spin (S = 2) (porphyrinato) iron (II) derivatives.

Compound	Fe–Np ^a^	Fe-XL ^b^	Fe–PN ^c^	Fe–PC ^d^	Reference
Compound with low spin (S = 0)					
[Fe^II^(TpivPP) (NO_2_)]	1.970 (4)	1.849 (6)	N.R ^e^	0.15	Nasri et al. (2004)
[Fe^II^(TpivPP) (1-MeHIm)_2_]	1.990 (2)	1.720 (1)	0.26	0	Scheidt and Frisse (1975)
[Fe^II^(TPP) (CN)_2_]^2-,^ ^f^	1.990	------	0	N.R ^e^	Li et al. (2008)
[Fe (TPP) (NO)] ^f^	2.001 (3)	1.717 (7)	0.21	0.21	Mazzeo et al. (2022)
Compound with high spin (S = 2)					
[Fe (TpivPP) (HCO_3_)]^-^	2.079 (3)	2.070 (4)	0.40	0.47	t.w.
[Fe (TpivPP) (O_2_CCH_3_)]^-^	2.107 (2)	2.034 (3)	0.55	0.64	Nasri et al. (1987)
[Fe (TpivPP) (NCO)]^-^	2.121 (2)	2.007 (3)	0.59	0.71	Dhifet et al. (2010)
[Fe (TpivPP) (SH)]^-^	2.102 (2)	2.312 (8)	0.52	0.55	Dhifet et al. (2009b)
[Fe (TpivPP) (2-MeIm)]^-^	2.110 (2)	2.002 (1)	0.52	0.65	Mandon et al. (1990)
[Fe (TpivPP) (OC_6_H_5_)]^-^	2.114 (2)	1.937 (4)	0.56	0.62	Schappacher et al. (1983)
[Fe (TpivPP) (SC_6_HF_4_)]^-^	2.077 (1)	2.370 (3)	0.42	N.R ^e^	Mashiko et al. (1981)

^a^ Fe–Np: the average equatorial iron–nitrogen pyrrole distance.

^b^ Fe–X_L_: the iron–axial ligand distance.

^c^ Fe–P_N_: the distance between the iron center and the mean plane of the four pyrrole N atoms.

^d^ Fe–Pc: the distance between the iron atom and the mean plane made by the 24-atom core of the porphyrin.

^e^ Value not reported.

^f^ TPP: tetraphenylporphyrin.

t.w.: this work.

The average equatorial Fe–N_p_ distance for the [Fe^II^(TpivPP) (HCO_3_)l^−^ ion complex is 2.079 (3) Å, and it falls within the range found for high-spin iron (II) porphyrins (2.072 Å–2.115 Å) (Mashiko et al., 1981). This provides stereochemical evidence that the synthetic compound adopts a high-spin configuration (S = 2). In order to accommodate the high-spin iron (II) atom, the porphinato cores undergo a significant radial expansion. This is well-illustrated by the long Fe–P_C_ and Fe–P_N_ distances shown by Fe (II) high-spin [Fe^II^(Porph) (X)]^−^ complexes (X is an anionic ligand). However, compounds with low spin (S = 0) present lower Fe–P_C_ and Fe–P_N_ distances.

Table 6 illustrates the distances (Å) for some hydrogencarbonato complexes.

**TABLE 6:** Selected bond length (Å) for some hydrogencarbonato complexes.

Complex	M–O^I^	C–O^I^	C–O^II^	C–O^III^	Reference
[Fe^II^(TpivPP) (η^1^-OCO_2_H)]^-^	2.086	1.220	1.242	1.386	This work
[Mg^II^(TPP) (η^1^-HCO_3_)]^-^ ^a^	1.958	1.268	1.251	1.327	Bhuyan et al. (2011)
[Zr^IV^(TPP) (η^1^-HCO_3_) (OPh)]^a^	2.258	1.252	1.283	1.494	Dhifet et al. (2025)

^a^ TPP, tetraphenylporphyrin.

The bond length values between the carbon atom and the two oxygen atoms O5 and O6 of the HCO_3_^−^ axial ligand of complex I are C65–O5 = 1.220 (4) Å and C65–O6 = 1.242 (5) Å. The second value is the longest C65–O7 distance of the HCO_3_^−^ ligand, which is longer than the C–O^I^ and C–O^II^ bond lengths for all species reported in Table 6. The non-planar conformation of the porphyrin core can be explained by the type of porphyrin and the number of coordination sites for the metal.

The HCO_3_^−^ anion presents two short optimized C–O distances of 1.247 Å and 1.263 Å and one longer C–O distance of 1.467 Å, for which the oxygen atom is linked to the H atom (Scheidt and Lee, 1987). The carbon atom is linked to the three oxygen atoms, and the longest C65–O7 distance is the one where the oxygen atom is bonded to the hydrogen atom. This is also the case for complex I, for which the HCO_3_^−^ axial ligand exhibits a unidentate-type coordination mode, and the C65–O5, C65–O6, and C65–O7 distances are 1.220 (4) Å, 1.242 (4) Å, and 1.386 (6) Å, respectively (see Figure 5).

**FIGURE 6:**
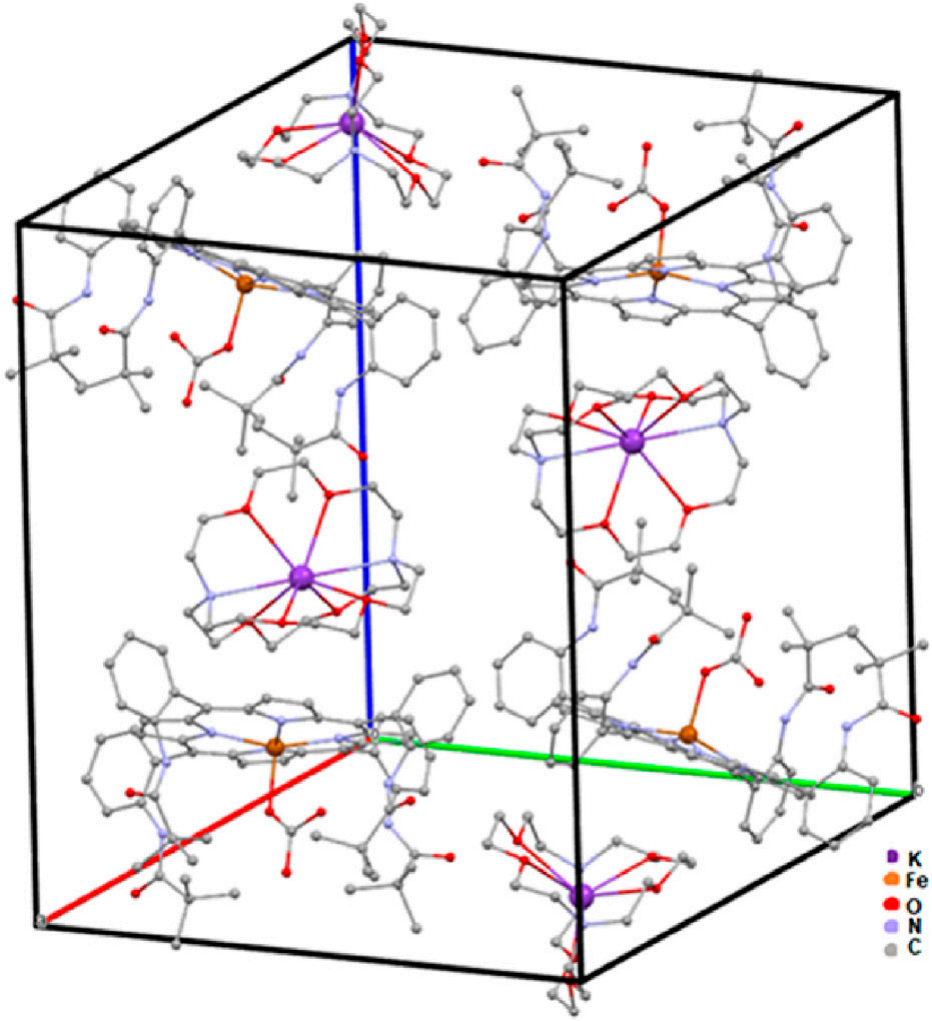
Representation of the crystal lattice of complex I.

Figure 6 illustrates the crystal lattice of complex I after the omission of disordered chlorobenzene.

**FIGURE 7:**
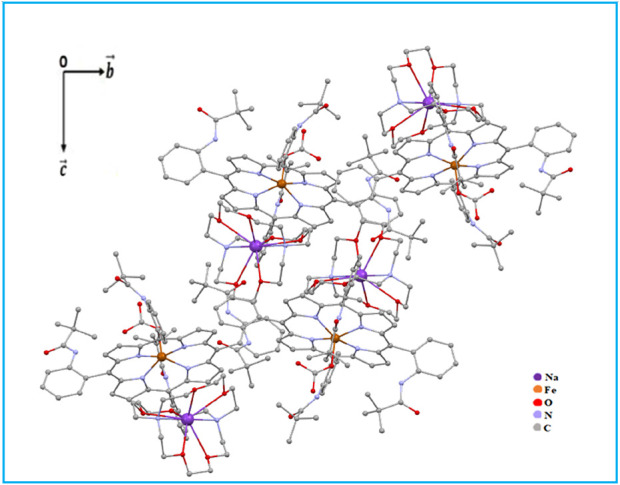
Representation of the crystal lattice of complex I.

A partial view of the crystal packing of the title compound plotted in projection along the [100] direction is illustrated in Figure 7.

**FIGURE 8:**
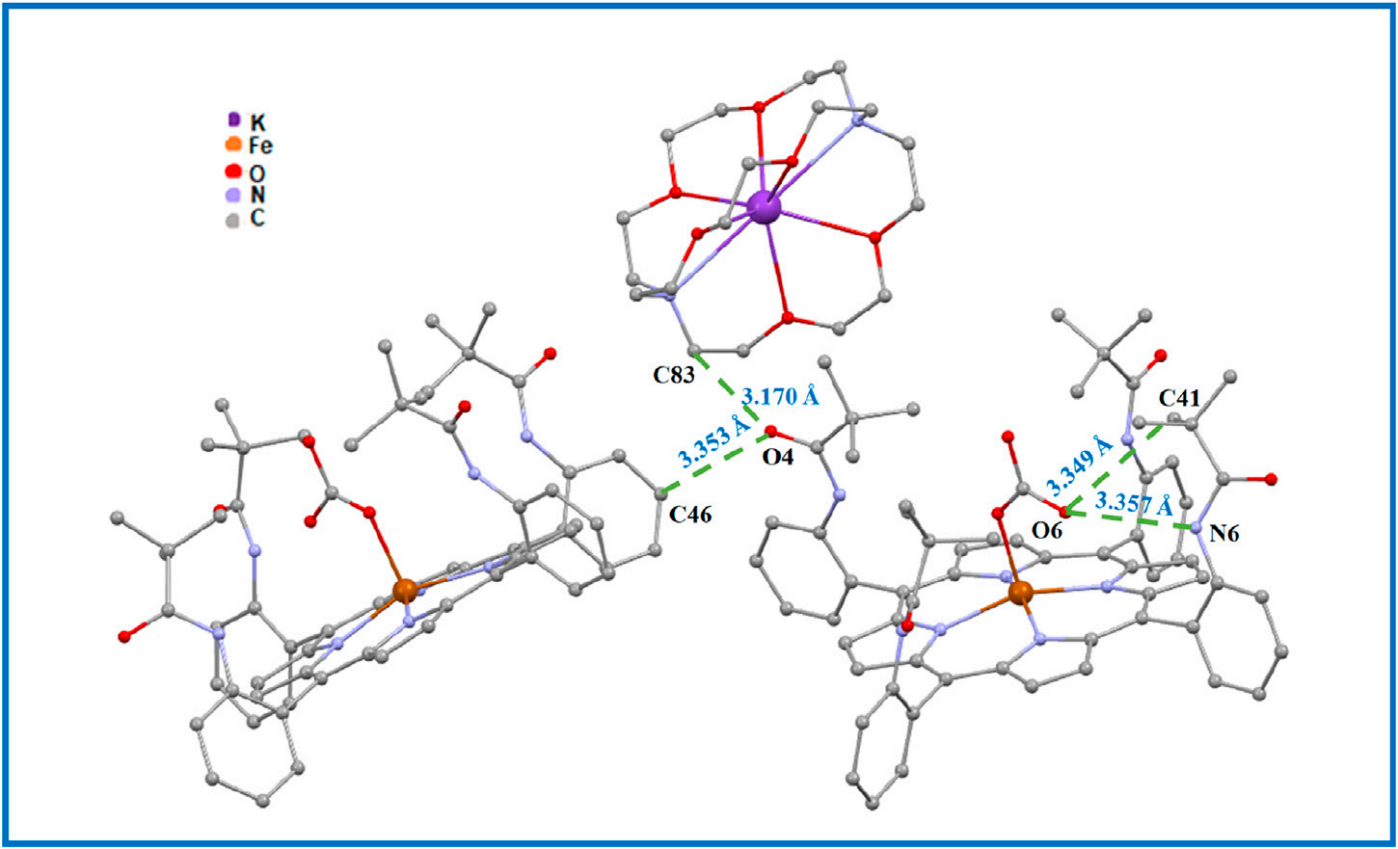
Drawing illustrating the C–H...O and N–H...O intermolecular interactions in complex I.

**FIGURE 9:**
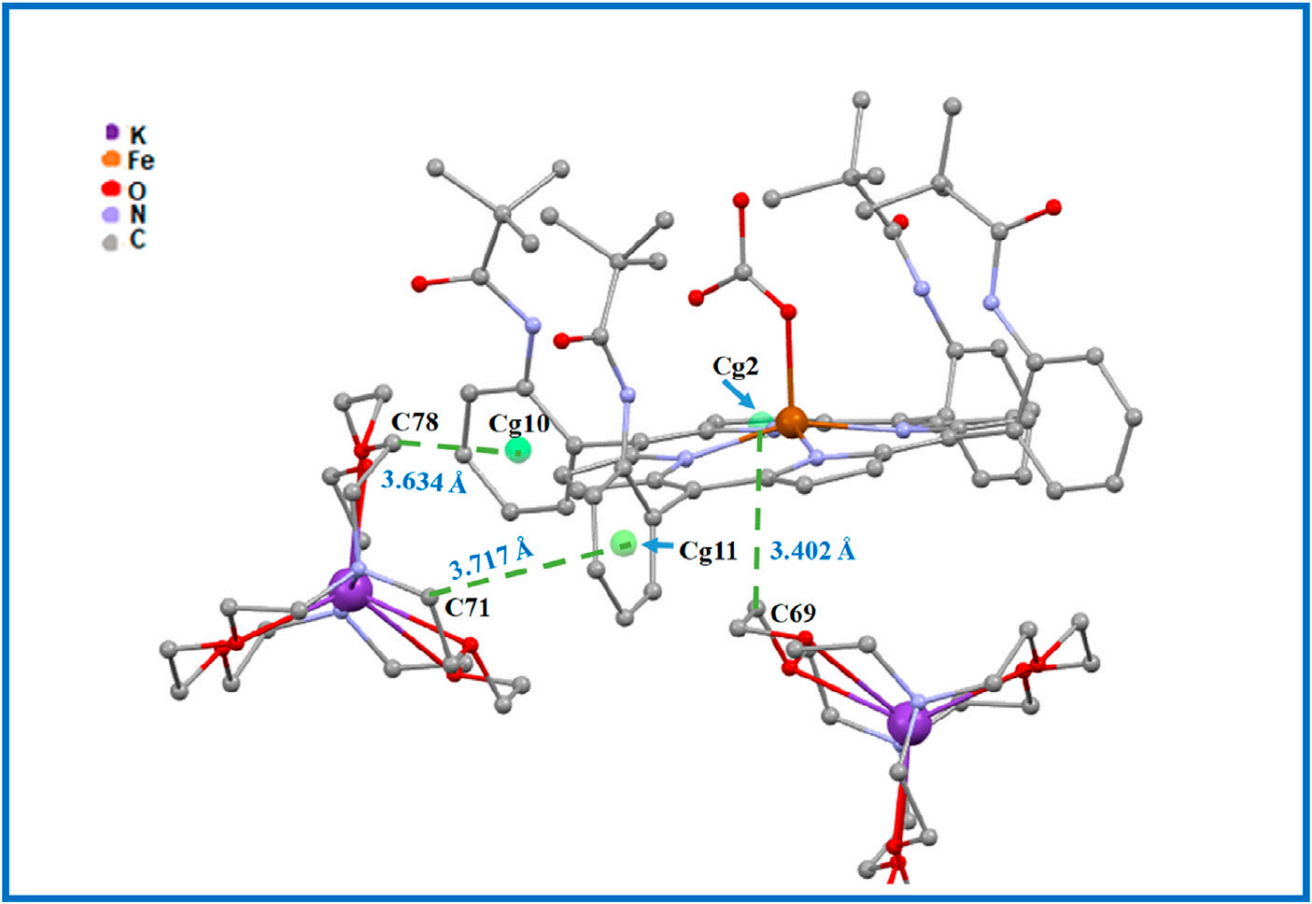
Drawing illustrating the C69–H69...Cg2, C78–H78B...Cg10 and C71–H71B...Cg11 intermolecular interactions in complex I.

In order to investigate the intermolecular interactions within the crystal packing of complex I, we used the PLATON program (Spek, 2015), as described above, to obtain a clear visualization of these intermolecular interactions (see Figures 8, 9; Supplementary Table S1).

The crystal packing of the title compound is stabilized by C–H … O, N–H … O, and C–H … *Cg* (*Cg* is the centroid of a phenyl or a pyrrole ring of the TpivPP porphyrinate) interactions, leading to a two-dimensional (2D) network. In the crystal packing of complex I, one [Fe^II^(TpivPP) (HCO_3_)]^-^ ion complex is linked to a neighboring [Fe^II^(TpivPP) (HCO_3_)]^-^ and [K (2,2,2-crypt)]^+^ species by seven intermolecular interactions (Figures 8, 9; Supplementary Table S1): (i) the oxygen atom O4 of a pivaloyl group of the first [Fe^II^(TpivPP) (HCO_3_)]^-^ ion complex is H-bonded to the carbon atom C46 of a second [Fe^II^(TpivPP) (HCO_3_)]^-^ ion complex, with a C46–H46 … O4 contact distance of 3.353 (9) Å, and is also H-bonded to the carbon atom C83 of a [K (2,2,2-crypt)]^+^ ion complex, with a C83–H83A … O4 contact distance of 3.170 (8) Å; (ii) the oxygen atom O6 of the HCO_3_^−^ axial ligand of the first [Fe^II^(TpivPP) (HCO_3_)]^-^ ion is weakly linked to the carbon atom C41 of the first [Fe^II^(TpivPP) (HCO_3_)]^-^ via the C41–H41B … O6 bond with a distance of 3.349 (12) Å and is H-bonded to the nitrogen atom N6 of the picket fence group, with a N6–H6 … O6 contact distance of 3.357 (7) Å; (iii) the carbon atom C69 of the [K (2,2,2-crypt)]^+^ ion is weakly linked to the Cg2 of the pyrrole group of the first [Fe^II^(TpivPP) (HCO_3_)]^-^ via the C69–H69A … Cg2 bond with a distance of 3.402 (7) Å; (iv) the carbon atom C71 of the [K (2,2,2-crypt)]^+^ ion is weakly linked to the Cg11 of the phenyl group of the first [Fe^II^(TpivPP) (HCO_3_)]^-^ via the C71–H71B … Cg11 bond with a distance of 3.717 (5) Å; and (v) the carbon atom C78 of the [K (2,2,2-crypt)]^+^ ion is weakly linked to the Cg10 of the phenyl group of the first [Fe^II^(TpivPP) (HCO_3_)]^-^ via the C78–H78B … Cg10 bond with a distance of 3.634 (7) Å.

**FIGURE 10:**
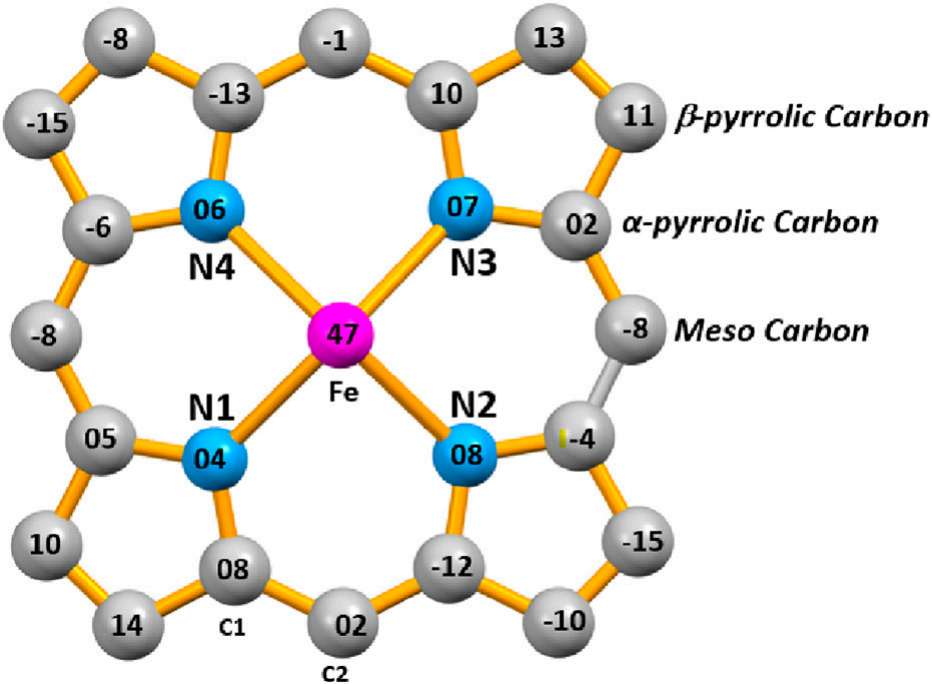
Porphyrin core in the [Fe^II^(TpivPP)(HCO_3_)]- illustrating the displacements of each atom.

The displacement of each atom of the porphyrin ring from the mean 24-atom core of the [Fe^II^(TpivPP) (HCO_3_)]^-^ is illustrated in Figure 10. This diagram shows a moderate ruffle and major distortion of the porphyrin macrocycle (Frisch et al., 2009).

### 3.4 Hirshfeld surface and intermolecular interaction analysis

**FIGURE 11:**
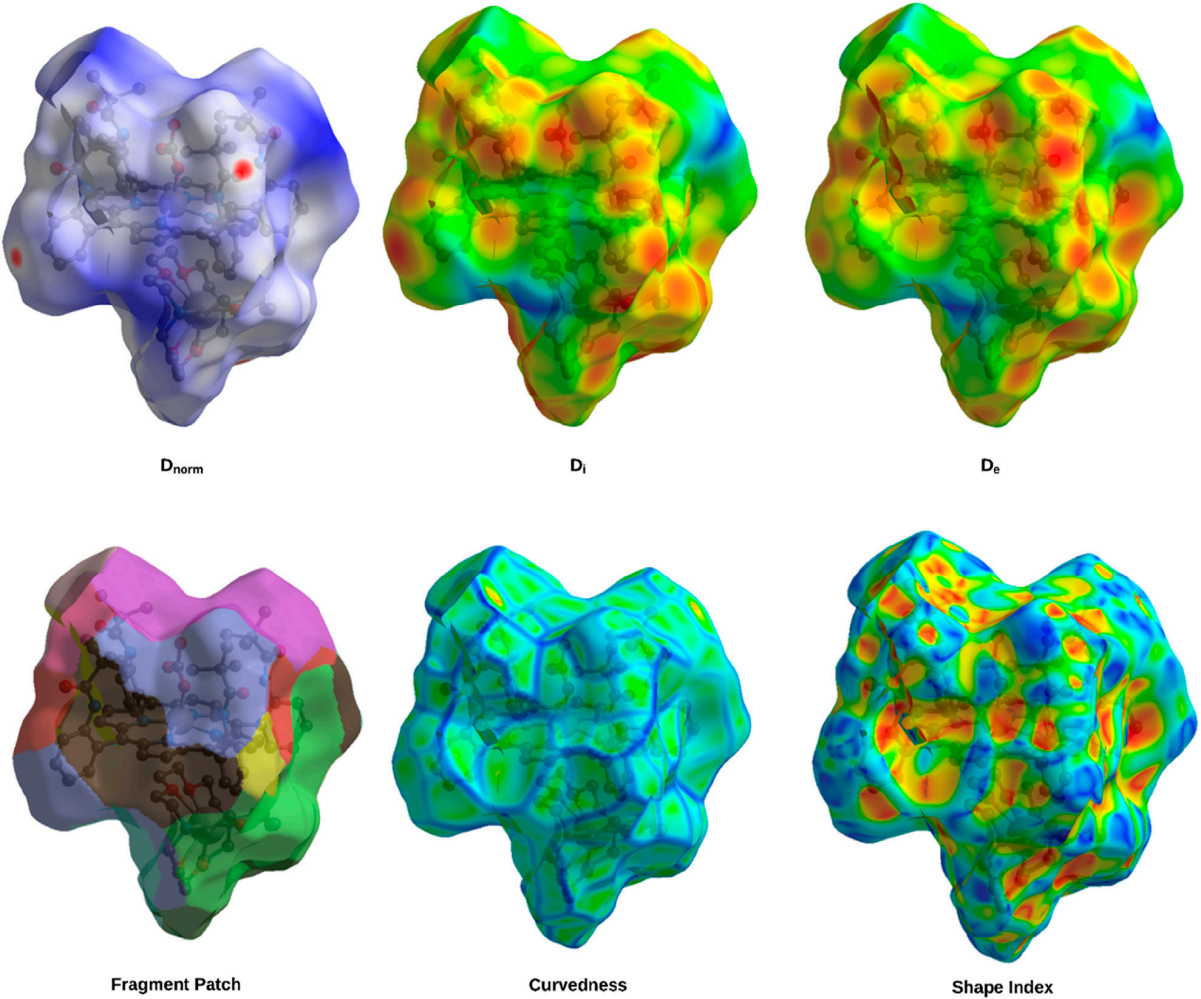
Hirshfeld surface maps of complex I highlighting the key intermolecular interactions via D_norm_, D_i_, D_e_, fragment patch, curvedness, and shape index.

Hirshfeld surface analysis was conducted on the iron metalloporphyrin complexes and their axial ligands to investigate the nature and extent of intermolecular interactions. Key surface properties, such as electron density deviations (d_norm_), shape index, curvedness, and others, were mapped to highlight contact regions. The results of this analysis are summarized in Figure 11.

Hirshfeld surface analysis was employed to characterize the nature and distribution of intermolecular interactions within the iron metalloporphyrin complexes. The d_norm_ mapping highlights distinct red spots on the surface corresponding to close contacts that are significantly shorter than the sum of the van der Waals radii, indicating strong interactions such as hydrogen bonds and π···π stacking. In contrast, large regions colored white and blue reflect contacts at typical or longer van der Waals distances, suggesting weaker or less frequent interactions. This pattern implies that the crystal packing is primarily governed by a limited number of strong, directional interactions rather than by widespread uniform contacts.

The complementary d_i_ (internal distance) and d_e_ (external distance) surfaces further reveal the spatial relationship between the atoms inside and outside the molecular surface. The red regions in these maps mark atoms involved in close intermolecular contacts, confirming the anisotropic nature of molecular packing, where specific sites dominate the crystal stabilization.

Analysis of the d_norm_ identifies functional groups contributing to intermolecular bonding: the red areas correspond to hydrogen bond acceptors (e.g., oxygen and nitrogen atoms), the blue regions mark hydrogen bond donors (hydrogen atoms), the yellow areas indicate hydrophobic contacts between nonpolar groups, and the green regions highlight aromatic or alkyl fragments involved in π–π or alkyl–π interactions. The purple–pink patches show polar or charged sites that are important for electrostatic interactions, all of which contribute synergistically to the stability of the molecular assembly.

The curvedness map distinguishes planar aromatic surfaces (low curvedness) that facilitate extensive π···π stacking from protruding substituents (high curvedness) involved in directional interactions, such as C–H···O/N hydrogen bonds and C–H···π contacts. Meanwhile, the shape index surface reveals alternating concave and convex patches characteristic of π···π stacking interactions, further emphasizing the significance of aromatic ring interactions in the crystal lattice. Together, these complementary Hirshfeld surface analyses highlight the importance of both strong localized interactions and broader dispersive forces in maintaining the stability of the crystal structure.

### 3.5 Fingerprint plot analysis and contact contributions

**FIGURE 12:**
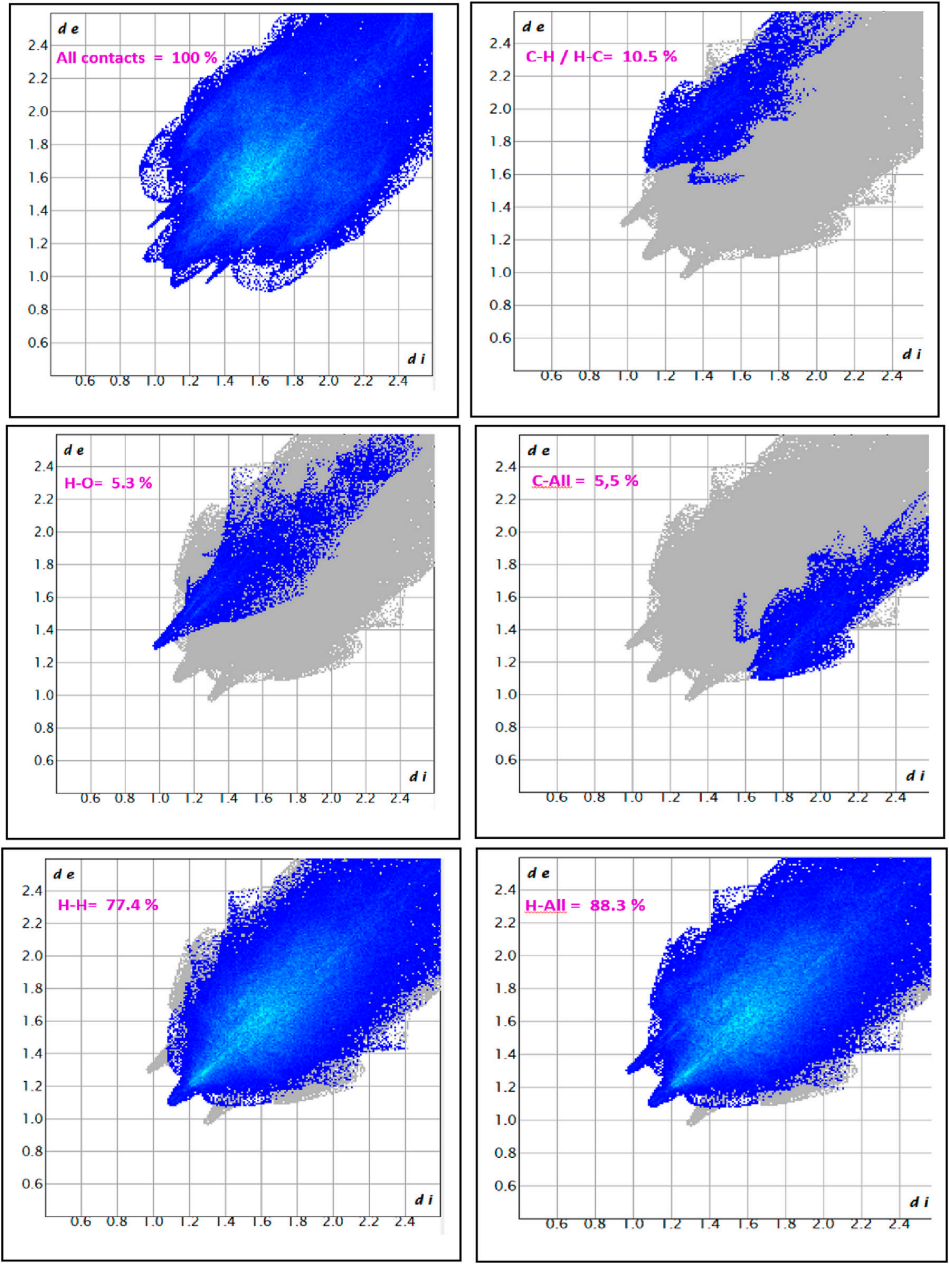
2D-fingerprint plot of complex I.

The two-dimensional fingerprint plots for complex I, shown in Figure 12, offer a comprehensive depiction of the intermolecular interactions within the crystal structure. The symmetrical distribution along the diagonal axis confirms the presence of a single, crystallographically unique molecular unit, with no contribution from additional solvent molecules or multiple independent moieties. The overall fingerprint captures 100% of all the contact interactions, with the densest regions being concentrated around d_i_ and d_e_ values between 1.0 Å and 2.2 Å, indicating the most frequent interatomic distances.

A closer inspection of individual contact types reveals that the H⋯H interactions are overwhelmingly dominant, contributing 77.4% of the total Hirshfeld surface. This interaction is characterized by a broad, continuous region in the plot, indicative of extensive van der Waals forces among hydrogen atoms, which play a central role in the stabilization of the crystal packing. The C–H⋯H–C interactions represent 10.5% of the contacts and exhibit a more localized and less intense distribution, suggesting a moderate contribution to the structural cohesion.

Hydrogen bonding is evidenced by the H⋯O contacts, accounting for 5.3% of the interactions. These appear as sharp, narrow spikes in the fingerprint plot and are a signature of strong and highly directional hydrogen bonds within the crystal lattice. Furthermore, C⋯all interactions contribute 5.5% of the total, displaying a scattered distribution that reflects weaker van der Waals interactions between carbon atoms and other non-hydrogen species. Finally, the cumulative H⋯all contacts constitute 88.3% of the total, reinforcing the overwhelming significance of hydrogen-involved interactions in the molecular assembly.

Collectively, these findings highlight the predominance of dispersive H⋯H contacts in the crystal architecture of complex I, with secondary contributions from C–H and hydrogen-bonding interactions, thereby outlining a packing environment driven primarily by hydrogen-related forces.

## 4 DFT-computational investigations of compound I

### 4.1 Optimized structure

DFT calculations were performed using the Gaussian 09 software package (Ismail et al., 2021). The structural optimization and subsequent property analyses were carried out on the cationic and anionic components of the synthesized complex. A hybrid basis set approach was employed to accommodate the varying atomic species within the complex: for light atoms (C, H, N, and O), the 6-31G (d,p) basis set was used, incorporating polarization functions to account for directional bonding and lone-pair effects. For the transition metal center (Fe), the LANL2DZ basis set and corresponding effective core potential (ECP) were used to efficiently treat relativistic and core-electron effects (Dennington et al., 2000). Geometry optimizations were conducted in the gas phase using the default convergence criteria, without imposing symmetry constraints, in order to allow full relaxation of the molecular framework, and the results are presented in Table 7 along with the experimental distances and angles. Frequency calculations were performed to confirm the nature of the stationary points and ensure the absence of imaginary frequencies, confirming the true minima on the potential energy surface.

**TABLE 7:** XRD and DFT optimized distances (Å) and angles (°) of the iron coordination polyhedron and the cation complex.

Iron coordination polyhedron					
Fe–N1	2.070 (3) 2.068	N1–Fe–N2	88.15 (13) 88.019	N1–Fe–O5	97.48 (16) 96.914
Fe–N2	2.055 (3) 2.059	N1–Fe–N3	157.33 (14) 157.286	N2–Fe–O5	104.65 (16) 104.708
Fe–N3	2.083 (3) 2.077	N1–Fe–N4	86.67 (13) 86.492	N3–Fe–O5	105.04 (16) 104.940
Fe–N4	2.098 (3) 2.073	N2–Fe–N4	157.90 (14) 158.080	N4–Fe–O5	97.32 (16) 97.493
Fe–O5	2.070 (4) 2.047	N2–Fe–N3	88.65 (12) 88.165	C65–Fe–O5	123.5 (4) 124.2
Hydrogencarbonato ligand					
O5–C65	1.219 (4) 1.215	O6–C65-O5	117.5 (6) 116.92837		
O6–C65	1.242 (4) 1.2301	O6–C65–O7	118.6 (5) 119.02380		
O7–C65	1.386 (5) 1.3795	O5–C65–O7	123.9 (5) 123.96558		
O7–HO7	0.8200 0.8161				
Potassium-cryptand-2,2,2					
K–N9	3.024 (4) 3.09869	K–O11	2.825 (3) 2.85908	N9–K–O9	123.86295 123.26927
K–N10	3.054 (4) 3.09893	K–O12	2.846 (3) 2.8128	N9–K–O10	120.20 (11) 120.46245
K–O8	2.801 (4) 2.8142	K–O13	2.786 (4) 2.76080	N9–K–O11	60.54 (11) 60.07627
K–O9	2.820 (3) 2.8319	N9–K–N10	179.84 (14) 179.85681	N9–K–O12	60.86 (12) 60.07769
K–O10	2.854 (3) 2.85259	N9–K–O8	59.72 (13) 60.09301	N9–K–O13	120.39 (12) 121.40715

The comparison between experimental X-ray crystallographic data and DFT-optimized geometries for complex I reveals a strong correlation, affirming the accuracy and reliability of the computational model. The Fe (II) center is coordinated in a distorted octahedral geometry by four nitrogen atoms from the ligand framework and one oxygen atom from the hydrogen carbonato ligand. The experimental Fe–N bond lengths range from 2.055 to 2.098 Å, while the corresponding DFT values fall within a narrow interval of 2.058 Å–2.078 Å. Similarly, the Fe–O5 bond length is 2.070 Å experimentally and 2.0466 Å in the optimized structure. These small deviations (generally <0.02 Å) indicate an excellent agreement between the experimental and calculated values.

The angular parameters around the Fe center further support the structural consistency between the experimental and theoretical data. Both the cis- and trans-angles are well-reproduced in the DFT model, with values ranging from ∼86.5° to ∼158° in both datasets. The most pronounced trans-angles, such as N2–Fe–N4 and N1–Fe–N3, show near-perfect agreement with deviations of less than 0.1°, confirming that the distorted octahedral coordination environment is accurately maintained in the optimized structure.

Analysis of the hydrogen carbonato ligand geometry shows similarly consistent results. The C–O bond distances obtained experimentally (1.219 Å–1.386 Å) are in close agreement with the DFT values (1.215 Å–1.379 Å), correctly reflecting the varying bond orders and partial delocalization expected within the CO_3_ group. The O–C–O bond angles also align well, with deviations that are generally under 1°, thus maintaining the expected trigonal planar arrangement around the central carbon atom.

The potassium–cryptand-2,2,2 subunit exhibits more flexibility due to the large ionic radius and encapsulating nature of the ligand. Nonetheless, the DFT results remain in excellent concordance with the crystallographic data. K–N and K–O bond lengths range from 2.76 to 3.09 Å in the theoretical model, matching the experimental range of 2.78 Å–3.05 Å. Furthermore, key angular parameters, such as N9–K–N10, display nearly ideal linearity in both datasets (179.84° experimental vs 179.85° DFT). Other K–N–O angles also remain within acceptable deviation, thus validating the computational description of the coordination environment within the cryptand cavity.

Collectively, these results confirm that the DFT-optimized geometry reproduces the experimental structure of complex I with high fidelity. The consistency observed in bond lengths and angles validates the use of the applied computational methodology for further analysis, including electronic structure calculations and studies of intermolecular interactions. Such a level of agreement supports the robustness of the theoretical approach in capturing both the local coordination geometry and the extended supramolecular features of the complex.

### 4.2 Molecular orbital and electrostatic potential analysis

The frontier molecular orbitals were analyzed to evaluate the electron density distribution and electronic behavior of the high-spin iron (II) complex. In this open-shell (S = 2) system, the singly occupied molecular orbitals (SOMOs) were considered instead of the conventional highest occupied molecular orbital (HOMO), which applies to closed-shell systems. The SOMO and the lowest unoccupied molecular orbital (LUMO) were visualized to highlight the spatial distribution and nodal characteristics of the frontier orbitals. These visualizations were generated using GaussView 6.0 (Suresh et al., 2022) based on spin-unrestricted DFT results, and they reflect the α-spin electron configuration relevant to the reactivity of the complex.

Additionally, the electrostatic potential (ESP) surface was computed from the DFT-optimized geometry to map regions of electron richness and electron deficiency across the molecular surface (Jacobsen, 2008). This analysis provides complementary insight into the potential reactive sites and zones of intermolecular interaction.

Altogether, these visual representations support the interpretation of electronic properties and ligand-field effects and aid in rationalizing the potential nucleophilic or electrophilic behavior within the molecular framework.

#### 4.2.1 HOMO/LUMO visualization

**FIGURE 13:**
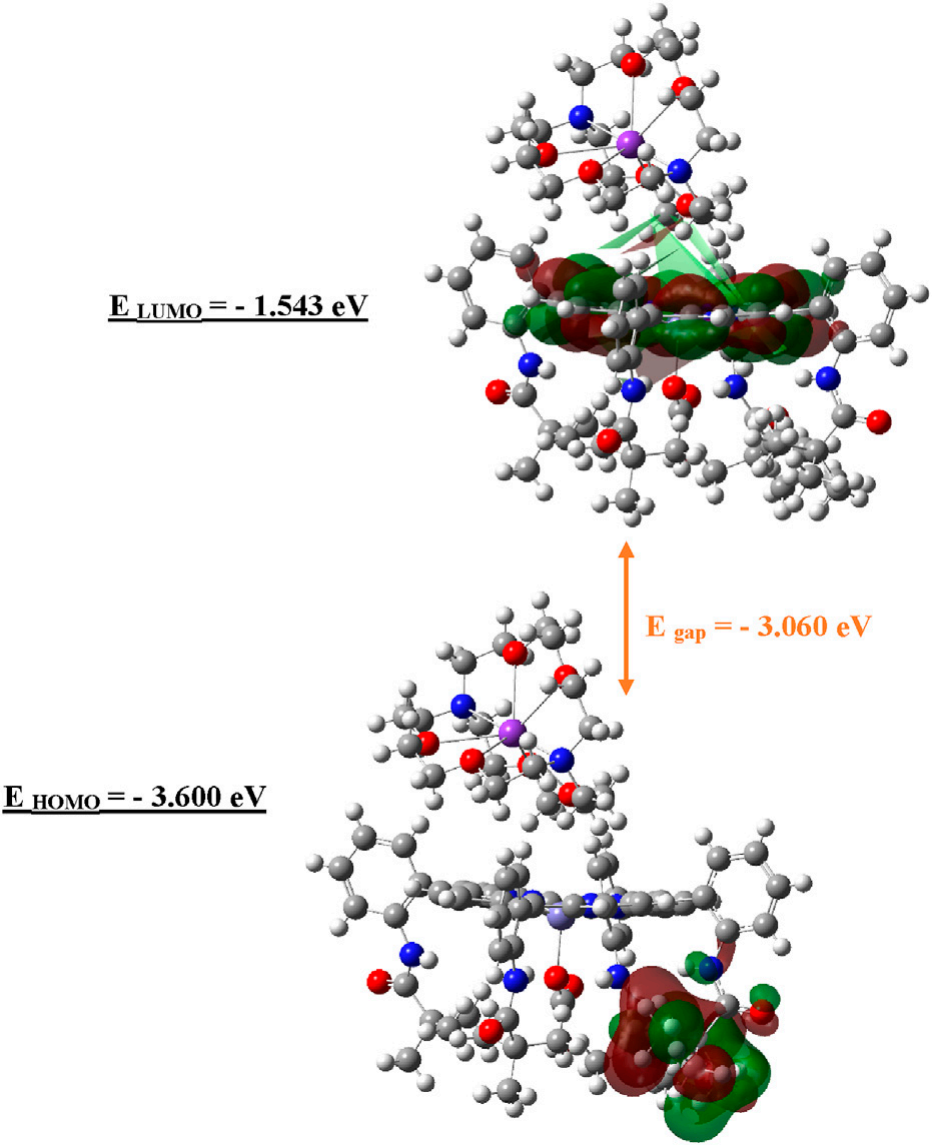
HOMO/LUMO visualization and frontal orbital energies of complex I. E _gap_ = - 3.060 eV. E _HOMO_ = - 3.600 eV. E _LUMO_ = - 1.543 eV.

To gain a deeper insight into the electronic behavior of the iron metalloporphyrin complexes, a frontier molecular orbital analysis was performed using spin-unrestricted DFT. Given the open-shell nature of the high-spin Fe (II) center (S = 2), particular attention was paid to the SOMOs, which replace the conventional HOMO concept used in closed-shell systems. The energies and spatial distributions of the SOMOs and the LUMOs are critical for evaluating the reactivity and stability of the complexes, especially in the context of oxo-transfer processes. The SOMO reflects the electron-donating capability, while the LUMO indicates the electron-accepting potential, both of which are essential parameters governing the catalytic activity of these systems. The results are presented in Figure 13.

As illustrated in Figure 13, the frontier molecular orbital (FMO) analysis reveals that the SOMO and LUMO are predominantly localized on the anionic part of the complex. Specifically, the LUMO exhibits a strong contribution from the Fe (II) center, highlighting its role in the complex’s electron-accepting behavior. The calculated energies of the SOMO and LUMO are −3.600 eV and −1.543 eV, respectively, corresponding to an energy gap of 2.06 eV. This theoretical gap is in close agreement with the experimentally determined optical band gap from UV–VIS spectroscopy (2.016 eV), lending further support to the semiconducting potential of complex I. The consistency between the theoretical and experimental data underscores the reliability of the computational model and reinforces the potential applicability of this compound in electronic or optoelectronic materials.

#### 4.2.2 Molecular electrostatic potential

**FIGURE 14:**
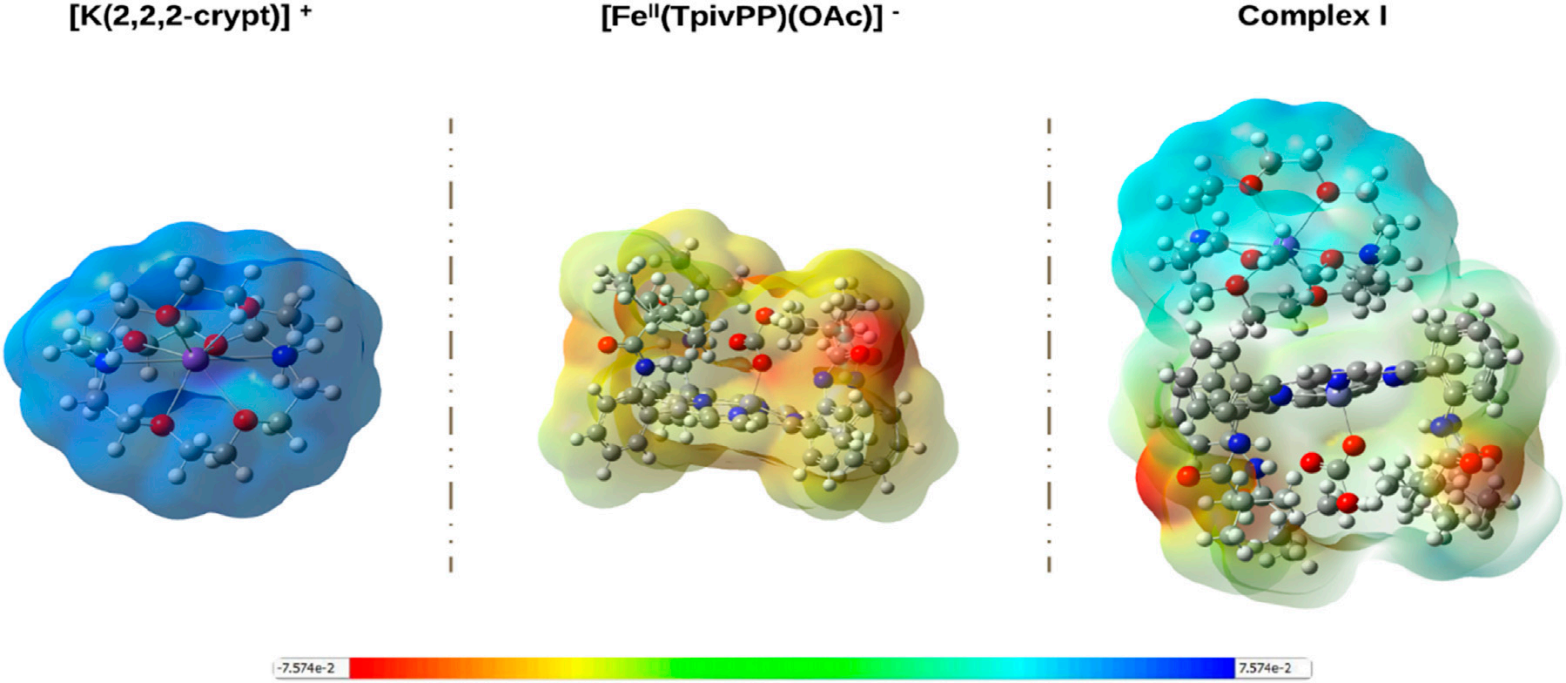
Electrostatic potential surface maps of the iron metalloporphyrin complexes. The color scale ranges from negative (red) to positive (blue) electrostatic potential.

The MEP surface, which is presented in Figure 14, offers a direct visualization of the charge distribution and potential reactive sites within the iron metalloporphyrin complex. Rather than serving as a purely qualitative image, the MEP reveals how the polarity and electrostatic landscape govern noncovalent interactions, complementing insights from the frontier molecular orbitals. Regions of negative potential (red/orange) indicate electron-rich domains that are prone to electrophilic attack, whereas regions of positive potential (blue/light blue) highlight electron-deficient areas that are susceptible to nucleophilic attack. This interplay between charge-rich and charge-poor regions plays a decisive role in dictating the preferred interaction sites in the supramolecular framework.

For the cationic [K (2,2,2-crypt)]^+^ fragment, the MEP map shows an almost uniform light blue coloration, reflecting a consistently positive potential (up to +0.125 a.u.). This is consistent with its net positive charge and suggests strong electrostatic attraction toward negatively charged species. In the crystal lattice, this would facilitate directional binding to the anionic metalloporphyrin unit, which stabilizes the ionic assembly through Coulombic interactions.

In contrast, the anionic [Fe^II^(TpivPP) (η^1^-HCO_3_)]^-^ fragment exhibits extensive red and orange areas that are particularly concentrated around the oxygen atoms of the η^1^-hydrogencarbonato ligand. These intense negative-potential regions (down to −0.129 a.u.) correspond to nucleophilic hot spots, representing the most probable sites for interactions with cationic species or electrophilic reagents. The complementary electrostatic profiles of the two fragments confirm that electrostatic attraction is the dominant driving force in complex stabilization, while the local potential distribution provides a map for understanding secondary hydrogen bonding or coordination interactions within the crystal.

### 4.3 ELF and LOL analysis

The ELF and LOL analyses were performed to visualize the electron localization within the compound. These functions provide a real-space representation of electron pairing and localization, offering insights into bonding patterns, lone pairs, and core electron regions (Lu and Chen, 2012; Zaki et al., 2024). Both functions were computed using the electron density derived from the optimized geometries, which were obtained at the DFT/B3LYP level with the dual basis sets LANL2DZ and 6-31G (d,p). The calculation of ELF and LOL, along with the generation of their respective 2D contour maps, was carried out using Multiwfn 3.4.1 [52].

**FIGURE 15:**
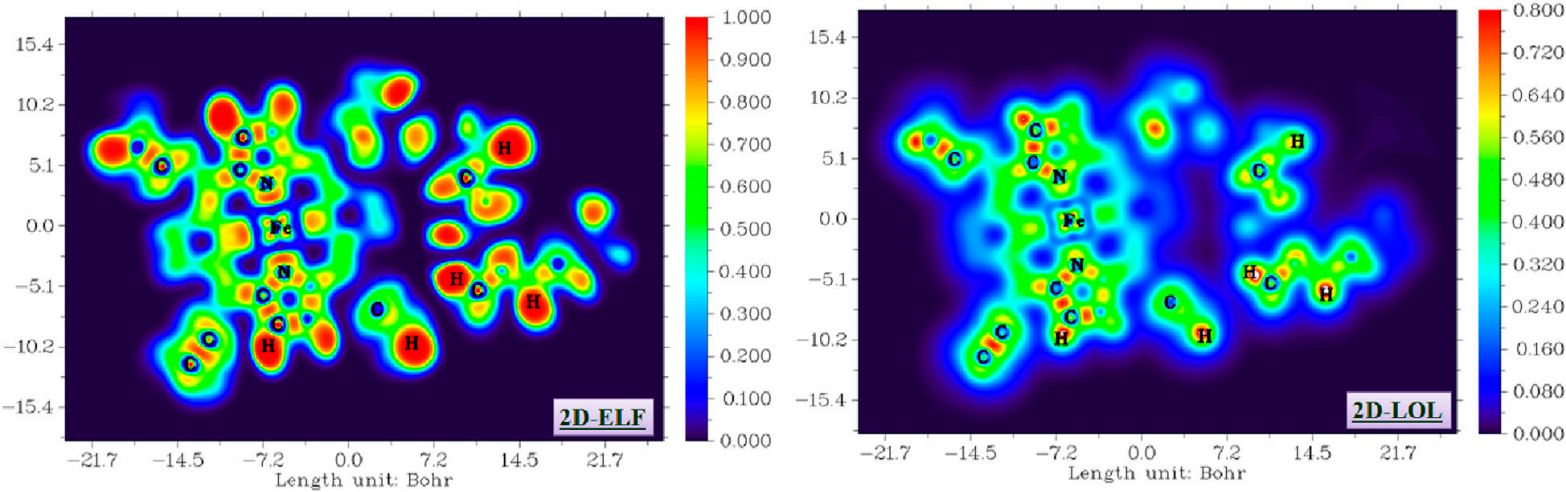
ELF and LOL iso-surface of the studied compound.

ELF and LOL analyses were performed to elucidate the detailed electronic structure and bonding characteristics of the title compound. These functions provide a quantitative measure of electron pairing and localization, enabling the clear delineation of covalent bonds, lone pairs, and core electron regions. The visualization of these localization domains offers critical insights into the nature of chemical bonds, the distribution of non-bonding electron density, and ultimately, the molecule’s overall stability and reactivity. Figure 15 presents a 2D representation of the LOL and ELF isosurfaces, projected onto the XY plane (Z = 0), with a color scale ranging from blue to red. It illustrates the delocalization and localization of electrons in each fragment of complex I.

High ELF/LOL values (approaching 1, red/yellow regions in Figure 15) are observed in the internuclear regions of C–H, C–C, and N–H bonds, confirming the strong covalent character. The ELF analysis clearly visualizes lone pairs on nitrogen and oxygen atoms, particularly around the hydrogencarbonato ligand, correlating with the high negative-potential areas identified in the MEP. This agreement between electrostatic and localization analyses provides a coherent picture of the electronic environment: electron-rich lone-pair regions not only govern local reactivity but also influence the hydrogen bonding patterns observed experimentally.

Importantly, the ELF/LOL patterns highlight delocalization within the porphyrin π-system, suggesting that the macrocycle contributes to the stabilization of electronic charge through conjugation. This delocalization, coupled with the distinct localization around donor atoms, rationalizes the orbital distribution observed in the HOMO–LUMO analysis and the moderate HOMO–LUMO gap. The synergy between MEP, ELF, and LOL results, therefore, provides a mechanistic understanding of how local electronic features influence both the molecular stability and potential reactivity, thus extending the discussion beyond a mere visual description of computational maps.

## 5 Conclusion

The five-coordinated complex I was prepared and characterized by electronic and IR spectroscopies and X-ray crystallography. The average equatorial iron-pyrrole N bond length (Fe–N_p_ = 2.079 (3) Å) and the distance between the iron and the 24-atom mean plane of the porphyrin ring (Fe–P_C_ = 0.466 (3) Å) are consistent with a high-spin (S = 2). This investigation confirms the type [Fe^II^(Porph) (X)]^-^, where Porph is the porphyrin picket-fence and X is the monodentate axial ligand. DFT calculations provided profound insights into the electronic structure and reactivity of the individual cationic and anionic components. The ESP maps clearly demonstrated complementary charge distributions, with a universally positive potential on the cryptand-2,2,2-encapsulated potassium cation and a predominantly negative potential on the anionic iron (II) complex, localized in particular on the oxygen atoms of the hydrogencarbonato ligand and the porphyrin. Furthermore, analysis of the frontier molecular orbitals revealed distinct electron-donating capacities for the anion and electron-accepting capabilities for the cation, underscoring the key orbital interactions driving their association. Finally, the Hirshfeld surface analysis for the entire complex, as studied by PLATON, elucidated the precise nature of the intermolecular interactions governing crystal packing, confirming a network of strong, localized contacts rather than a uniform distribution, which is crucial for the observed three-dimensional crystal architecture and stability. These combined experimental and computational approaches thus offer a comprehensive understanding of complex I, ranging from its fundamental electronic properties to its solid-state organization.

## Data Availability

The original contributions presented in the study are included in the article/Supplementary Material, further inquiries can be directed to the corresponding authors.
