# Supplementary material for: Comprehensive synthesis and characterization of a novel Fe–porphyrin complex: crystal structure, spectroscopic investigations, Hirshfeld surface analysis, and computational modeling (DFT, QTAIM-NCI/ELF)
Source: Front Chem. 2025 Sep 15;13:1666671. doi: 10.3389/fchem.2025.1666671 (PMC12476999; doi:10.3389/fchem.2025.1666671)
Supplement: Supplementary file 1 [file DataSheet1.pdf]

# Comprehensive Synthesis and Characterization of a Novel Fe-Porphyrin Complex: Crystalline Structure, Spectroscopic Investigations, Hirshfeld Surface Analysis, and Computational Modeling (DFT, QTAIM-NCI/ELF)

**Mondher Dhifet[a, b], Lelfia Guelmami[c, d], Khadija Zaki[e], Imen Zghab[f *], Abdelouahid Sbai[g], Bouzid Gassoumi[h*]**

[a] Laboratory of Physico-Chemistry of Materials (LR01ES19), Faculty of Sciences of Monastir, avenue of Environment, 5019 Monastir, Tunisia.

[b] University of Gafsa, Faculty of Sciences of Gafsa, Tunisia.

[c] Laboratory of Functional Physiology and Valorization of Bio-Resources (UR17ES27) at the Higher Institute of Biotechnology of Beja (ISBB), University of Jendouba, Tunisia.

[d] University of Jendouba, National Institute of Technology and Sciences of Kef, Tunisia.

[e] Laboratory of Molecular Chemistry and Natural Substances, University of Moulay Ismail, Faculty of Sciences, Meknes, Morocco.

[f] Department of Physical Sciences, Chemistry Division, College of Science, Jazan University, P.O. Box. 114, Jazan 45142, Kingdom of Saudi Arabia.

***Corresponding authors:** gassoumibouzid2016@gmail.com (B. Gassoumi), zghab@jazanu.edu.sa (I. Zghab)

**Contents**

1. Synthesis of starting products...……………………………………………….……………….2

2. UV/Vis spectroscopy…………………………………………………..……………………4

3. IR spectroscopy……………….…………………………………….…………………….5

4. X-ray molecular structure of complex **I**…...…………………………….………..........….......6

## 1. Synthesis of starting products

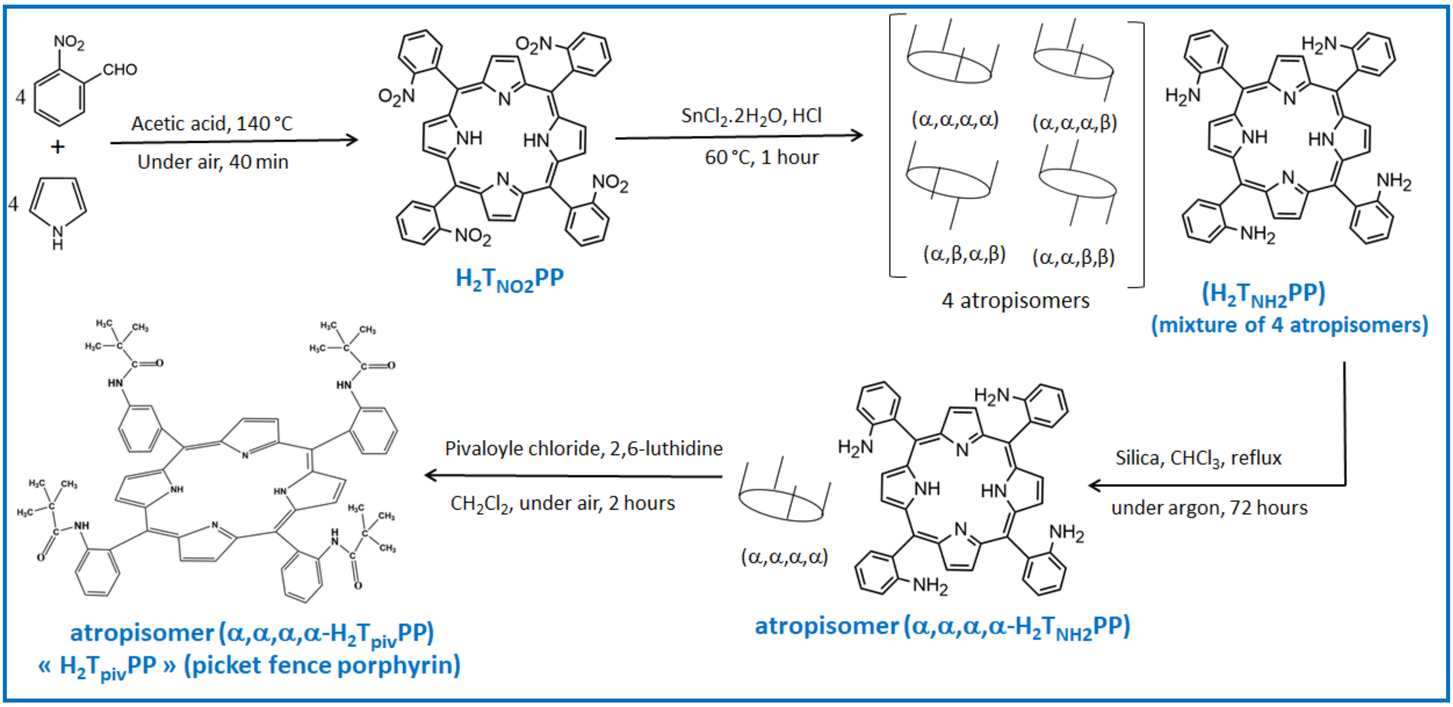


**Scheme S1**. Scheme of the preparation of the picket fence porphyrin ($H_2TpivPP$).

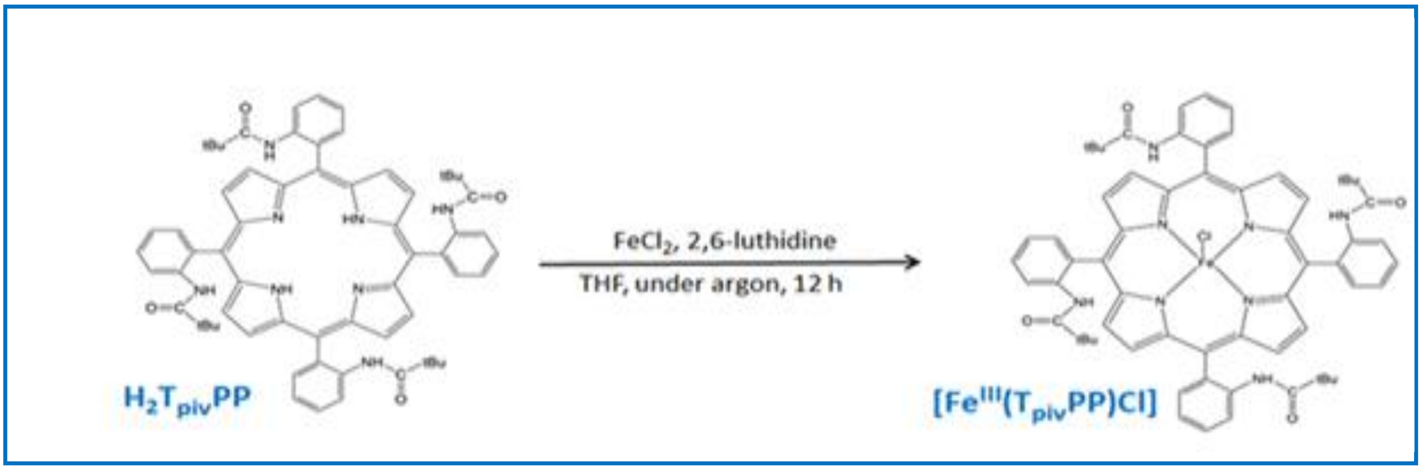


**Scheme S2**. Scheme of the preparation of the complex $[Fe^{III}(TpivPP)Cl]$.

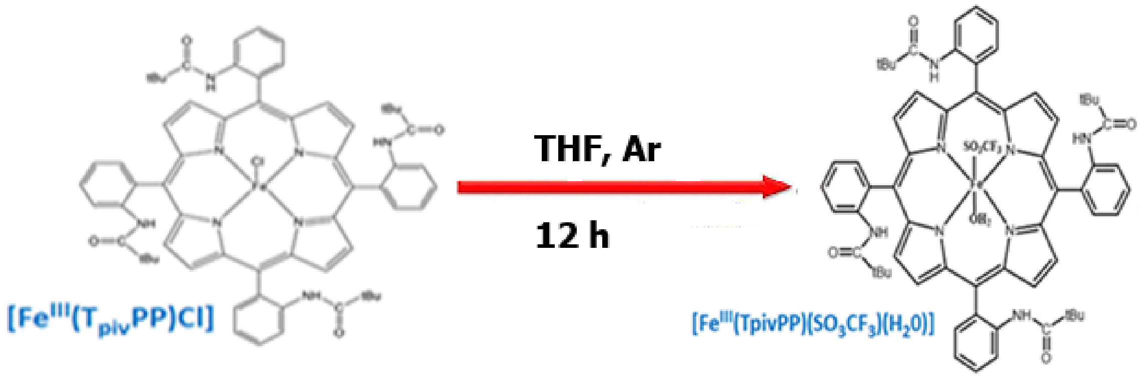


**Scheme S3**. Scheme of the preparation of the complex [Fe$^{III}$(TpivPP)($SO_3CF_3$)($H_2O$)].

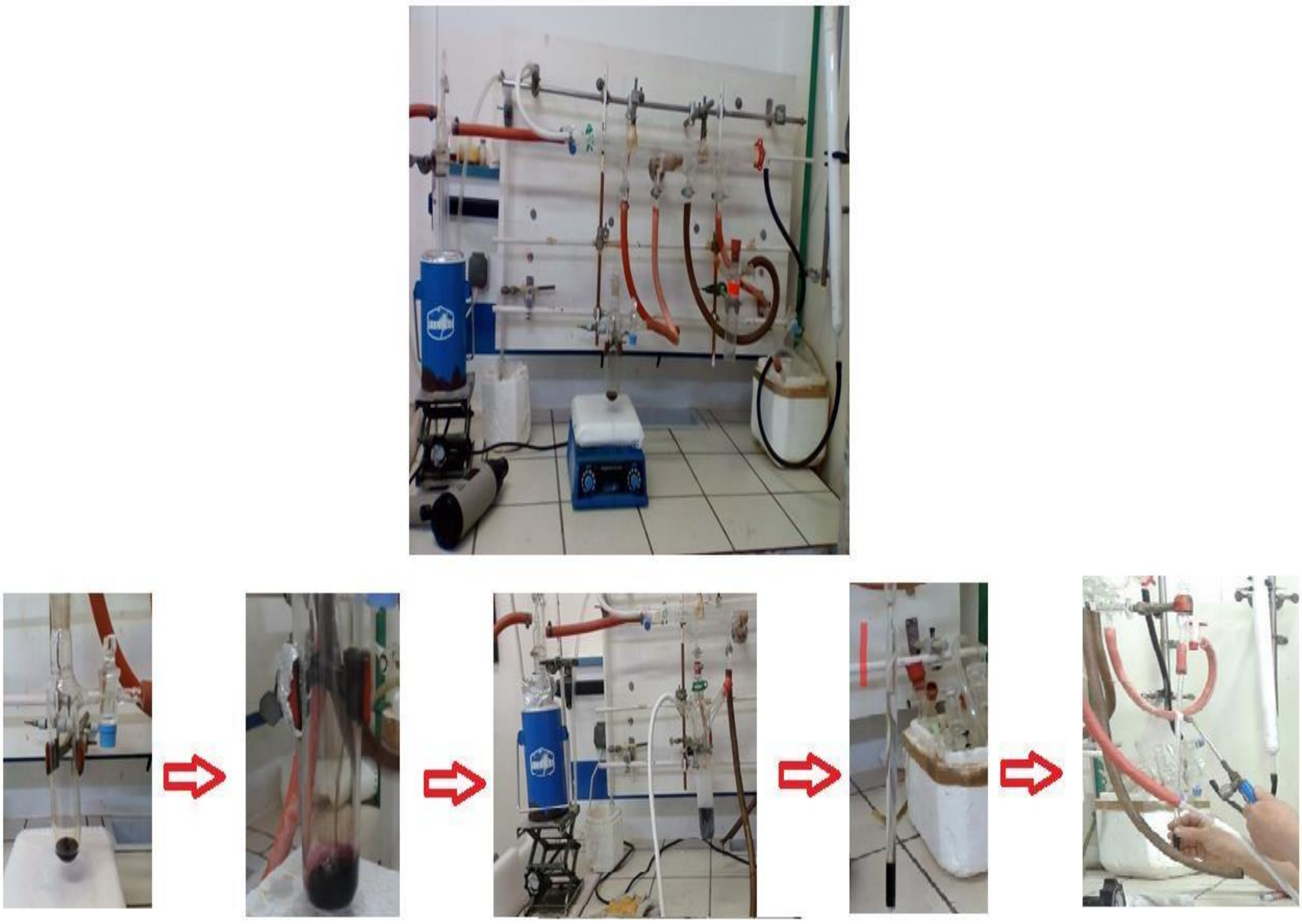

**Figure S1**. Different steps in the preparation of complex **I**.

## 2. UV/Vis spectroscopy

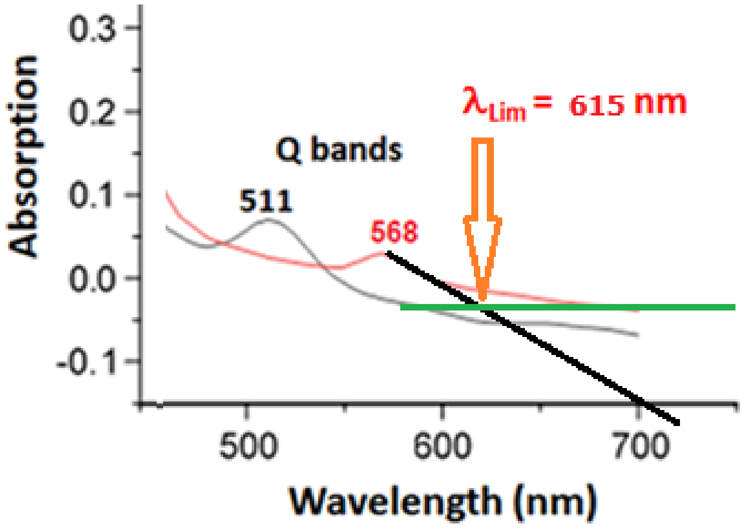


**Figure S2**. Determination of the $\lambda_{Lim}$ of complex **I**.

## 3. IR spectroscopy

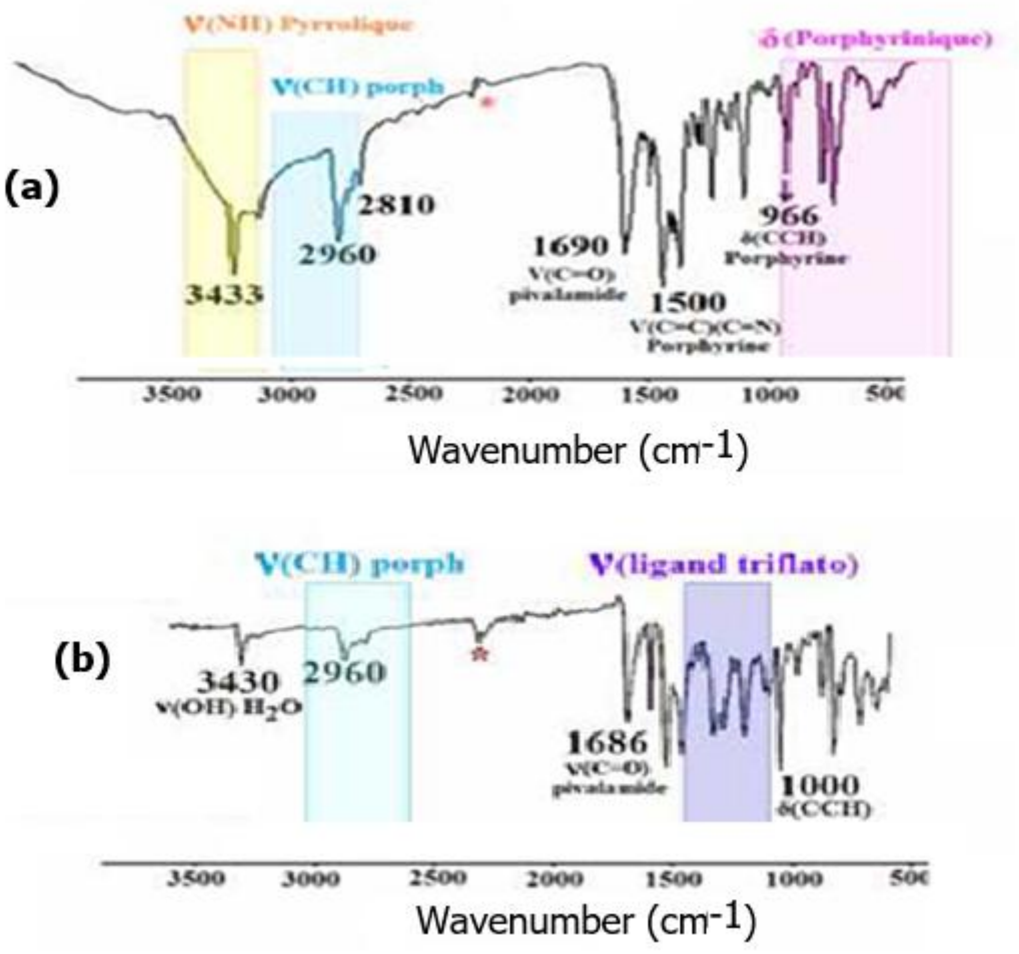


**Figure S3.** IR spectra of the free porphyrin $H_2$TpivPP **(a)** and the [$Fe^{III}$(TpivPP)($SO_3CF_3$)($H_2O$)] **(b)**.

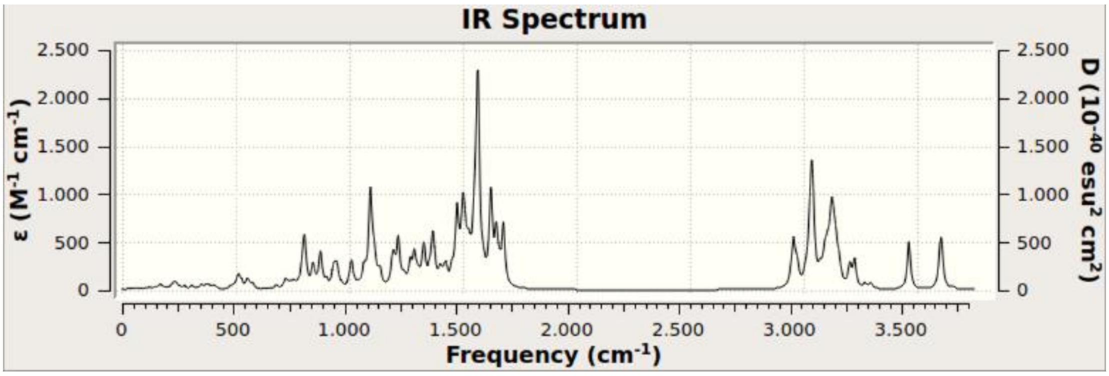


**Figure S4.** FT-IR spectrum of the complex **I** calculated at the DFT/B3LYP-D3/lanL2DZ level of theory.

## 4. X-ray molecular structure of complex I

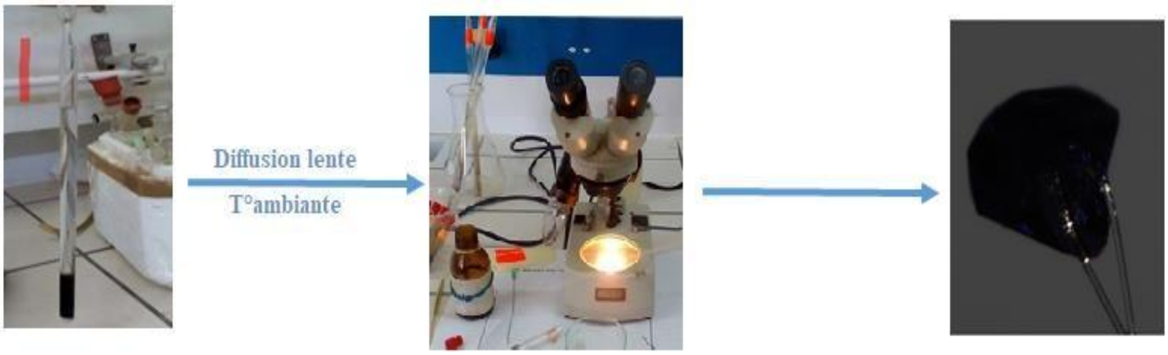


**Figure S5**. Steps in preparing a crystal for X-ray diffraction.

**Table S1**. Selected intermolecular interactions for complex **I**.

| D –H···A[a] | Symmetry of A | D···A (Å) | D–H···A (°) |
|---|---|---|---|
| N6-H6…O6 | x,y,z | 3.357(7) | 175 |
| C41-H41B…O6 | x,y,z | 3.349(12) | 152 |
| C46-H46B…O4 | -1/2+x,3/2-y,-1/2+z | 3.353(9) | 128 |
| C83-H83A…O4 | 1/2+x,3/2-y,-1/2+z | 3.170(8) | 141 |
| C69-H69B…Cg2[b] | x,y,z | 3.402(7) | 129 |
| C56-H56…Cg9[c] | 3/2-x,1/2+y,1/2- z | 3.784(5) | 162 |
| C78-H78B…Cg10[d] | 1-x,2-y,-z | 3.634(7) | 136 |
| C71-H71B…Cg11[e] | 1-x,2-y,-z | 3.717(5) | 137 |

[a] :D = donor atom and A = acceptor atom.
[b] Cg2 = Center of gravity of ring J (Plane number above N2 --> C6 --> C7 --> C8 --> C9).
[c] Cg9 = Center of gravity of ring J (Plane number above C21 --> C22 --> C23 --> C24 --> C25 --> C26).
[d] Cg10 = Center of gravity of ring J (Plane number above C32 --> C33 --> C34 --> C35 --> C36 --> C37).
[e] Cg11 = Center of gravity of ring J (Plane number above C43 --> C44 --> C45 --> C46 --> C47 --> C48).
